# Investigating the Influence Relationship Models for Stocks in Indian Equity Market: A Weighted Network Modelling Study

**DOI:** 10.1371/journal.pone.0166087

**Published:** 2016-11-15

**Authors:** Biplab Bhattacharjee, Muhammad Shafi, Animesh Acharjee

**Affiliations:** 1 School of Management Studies, National Institute of Technology, Calicut, Kerala, India; 2 Department of Biochemistry, Sanger Building, University of Cambridge, Cambridge, United Kingdom; East China University of Science and Technology, CHINA

## Abstract

The socio-economic systems today possess high levels of both interconnectedness and interdependencies, and such system-level relationships behave very dynamically. In such situations, it is all around perceived that influence is a perplexing power that has an overseeing part in affecting the dynamics and behaviours of involved ones. As a result of the force & direction of influence, the transformative change of one entity has a cogent aftereffect on the other entities in the system. The current study employs directed weighted networks for investigating the influential relationship patterns existent in a typical equity market as an outcome of inter-stock interactions happening at the market level, the sectorial level and the industrial level. The study dataset is derived from 335 constituent stocks of ‘Standard & Poor Bombay Stock Exchange 500 index’ and study period is 1^st^ June 2005 to 30^th^ June 2015. The study identifies the set of most dynamically influential stocks & their respective temporal pattern at three hierarchical levels: the complete equity market, different sectors, and constituting industry segments of those sectors. A detailed influence relationship analysis is performed for the sectorial level network of the construction sector, and it was found that stocks belonging to the cement industry possessed high influence within this sector. Also, the detailed network analysis of construction sector revealed that it follows scale-free characteristics and power law distribution. In the industry specific influence relationship analysis for cement industry, methods based on threshold filtering and minimum spanning tree were employed to derive a set of sub-graphs having temporally stable high-correlation structure over this ten years period.

## Introduction

In the past decades, there have been substantial empirical contributions focusing on investigating the socio-economic systems in the framework of complex network analysis [1–9]. Even though the concept of complex network analysis is not recent, studies in past [10] have emphasized the capability of this method to understand the principal attributes of interacting systems by examining the complete relational structure among agents and at the same time neglecting their detailed nature. For the better understanding of the complex systems under study, one should take into account the information concerning the intricate nature and strength of the underlying interactions among agents. In the context of an equity network, measuring the nature, direction and quantification of influence inherent in the underlying interactions between stocks will lead to better investment decisions.

These days the socio-economic systems possess higher degrees of interconnectedness and have a dynamic trend ingrained in their behavioural characteristics. In such scenarios, it is very much perceived that within a system framework, influence is a complex force that has a governing function in impacting the dynamic changes and behaviours of different entities constituting the system. As a result of the compelling capacity of influence, the transformational change of one entity within a system framework has a substantial effect on the other entities in the system. In general, the mining & characterization of influential nodes in financial system can be of great significance in early detection of risky set of elements in the system (which may include, banks, financial institutions, scripts listed in equity markets, important country-specific indices etc.) & further minimization of the impact of risk spread to different financial securities [11,12]. To illustrate the power of influence in global markets one can cite the examples of the cascading fall of the entire global financial system due to the bankruptcy of Lehman Brothers caused by the Subprime Mortgage Crisis originating in the US. Other examples include the sovereign debt risks in Portugal and Greece which resulted in subsequent turmoil in the global financial markets.

In the complex networks structure, interactions have customarily been treated to be binary in nature, indicating that two states can exist, one state being the two nodes interacting with each other, and therefore connected and second state being the two nodes are not interacting with each other hence not connected [13,14]. For imposing such binary interaction rules, there is a necessity of formulating threshold levels for interaction strengths; so that all interactions below those levels are considered to be eliminated. Despite the fact that this course of action is a reasonable first approximation step, the thresholding process can still drive a high magnitude of information loss in the system. Hence, a natural step forward is to construct a weighted network where weights are assigned on each of the interacting links, expressing their respective strengths of interactions [14]. Weighted network analysis has been used in several studies in past for identification of influential nodes and quantification of their interactive strengths. Some of them include the following: examining the flux movement in transportation related network, for instance the air traffic networks and Internet networks [15, 16]; analysis of the rate of turnover of molecules through a metabolic pathway in cellular systems [17,18, 19, 20]; study of the statistical properties of trading activity network existent in stock exchanges in developing economies [21,22,23]; investigation into the degree distribution, node distribution and weight distribution of the world investment networks[24] and lately for analysis of the dynamics of stock market networks [25,26,27].

In the context of stock market networks, several studies have employed weighted network models for characterising the behaviour of interacting agents [13–14,28–35]. The need for identification of influential agents in complex equity network is well justified from the study authored by Goyal and Van der Leij [36], which revealed that the topological structure of the financial network has an important role in determining the contagion at the time of crisis. A shock hitting a node with high levels of influence will result in quick transmission to the whole network and can accordingly trigger a systemic level crisis, whereas, a shock hitting a low influential node generates no particular issues to the system stability. This observation can be very critical from the perspective of international equity investors since with large likelihood one can evaluate the behaviour of equity market just by following the movement of few highly influential stocks [37]. For identification of influential nodes in a network, proper measures of influence strength are needed; some of the proposed measures of influence strength are discussed below.

Several studies have proposed different measures for quantification of influence in a weight network framework. The seminal study by Kim et. al. in the year 2002 proposed the first quantitative measure of influence in an equity network [37]. In this study, the influence-strength (IS) is stated as the summation of the weights of the edges that is incident on a given node. In addition to this, the study also observes that the probability distribution of influence strength follows a scale-free behaviour. Following this study, many other studies have proposed a new or modified version of the earlier definition of influence strength. Some of the studies define strength in the following manner: modification of Kim et. al. definition with incorporation of market capitalization [38]; strength as a sum of inter-node weights in a shortest path length [39]; strength as a sum of the inverse of sub-dominant ultrametric distances of a given Minimum Spanning Tree [40]; network centrality metrics as measure of strength [41]; strength computation based on PageRank algorithm [42] and cointegration networks[43].

The influence strength measure has found application in various financial markets such as, analysis of energy derivative markets network [29]; trading networks reflecting the relationship between stock seller and buyer[31]; international trade & financial integration networks[32]; international financial network[20]; network of ‘overnight money market’ segment of Italy[33]; network based on international real estate securities market[34]; global economic network [35]; and in identification of manipulated stocks in Chinese equity markets[30]].

The earlier empirical studies on equity networks, that have relevance for our current work, include those focusing on: Chinese equity network [44]; network formed of 32 country specific indices [45]; network formed of 1065 scripts listed in Hong Kong Exchange[46]; network formed of 116 New York Stock Exchange-traded scripts constituting the S&P 500 index[14]; network formed of 83 country specific indices.[47]; Polish equity market[48] and the network formed by scripts listed in KOSPI (Korea Composite Stock Price Index) 200 companies of Korean stock exchange[49].

In a typical stock market scenario, the movement of stocks depends on multiple factors such as, news releases on future estimated earnings and historical earnings and profits, dividend announcements, new product introduction or recall of substandard products, winning of new large government or MNC contract, layoffs of employees, news about expected takeover or mergers of firms, news regarding change in top management and recruitment of top professionals in CXO roles, accounting errors or scandals [50]. In addition to these factors, there lies three different types of influence behavioral traits among stocks, (i) movement of price of one stock influencing the price movement of another stock; (ii) movement of price of one stock influencing the price movement of group of stocks and vice versa; and (iii) movement of price of a group of common/related/diverse stocks influencing the price movement of another group of common/related/diverse stocks. The influence of movement of stock price on the other stock may be immediate (within the same day), delayed (next day or in a week), or long term (over a month). The conceptual model of influence relationship existent in a typical equity market is explained in later sections. The inherent influence of stock of one firm on stock(s) of other firm(s) is due to some of these factors: common industrial linkage (direct buyer-seller relationship in a vertically integrated supply chain), impact of common domestic or international macroeconomic changes, common promoter group, FII buying & selling pressure, mergers & acquisitions of companies, and behavioral investing in a bullish or bearish market.

Taking all the above factors into consideration, we can estimate that a simple influence of one stock on another is a multi-factorial complex phenomenon. To elucidate the nature and measure of this influence relationship, a complex weighted network based approach forms the appropriate strategy. In this paper, we exploit directed weighted network models to perform three layers of influence relationship networks analysis: global equity network, sector-specific networks, and industry specific network.

This study attempts to answer several interesting questions such as: Can the influence between two stocks be quantified? What is the correct measure of quantification? Which stocks have the highest influence over Indian equity market in last ten years? Is the influence of stock over a network dynamic or static? If the influence is a dynamic attribute, what is its temporal pattern over past ten year’s period? Which stocks has the highest influence in a particular sector? Can any economic reason be attributed to the consistent influence of stock in a sector? Which industrial segments have high levels of influence in a given sector? Can any economic reason be attributed to the consistent influence of particular industry segments in the sector?

The rest of the paper is organized as follows. The next section explains the influence relationship conceptual framework, which is followed by the description of the methodology employed for influence strength analysis at three hierarchical levels. A discussion of the results obtained from influence strength analysis follows. The last section provides the conclusive remarks and the further scope of the study.

## Materials & Methods

### Data

In the current study, we have taken stocks constituting the ‘Standard & Poor Bombay Stock Exchange 500 Index’(S&P BSE 500) as a representative of the Indian equity market. The S&P BSE 500 index gives a representation of approximately ninety-three percent of the entire market capitalization of all the stocks traded on Bombay Stock Exchange. S&P BSE 500 represents all the twenty major industrial segments of the economy. The study period consists of 1^st^ June 2005 to 30^th^ June 2015. The constituent scripts of S&P BSE 500 which had Initial Public Offering after 1^st^ June 2005 were excluded from our study dataset because of unavailability of continuous historical data of the ten years of the study period. It resulted in the inclusion of 335 stocks of companies listed in S&P BSE 500 Index in our study dataset. The daily stock closing price data, which were adjusted for historical splits and dividends announcements, for these 335 companies were retrieved from Bombay Stock Exchange historical reports for the period 1^st^ June 2005 to 30^th^ June 2015. In cases where the price value of the stock is found to be missing for a specific day/days, an issue that is very prevalent with datasets from emerging economy’s financial markets, it is considered that no was trading activity conducted on that particular day/days i.e. the price remained unchanged to the previous trading day. In the study dataset, there are 2489 data points (trading days) representing closing price for ten years duration. For the current study, we download the values of market capitalization for these scripts for a specific day in July 2015 and consider this as the representative set of values for the entire sample.

### Rolling window approach

The rolling window approach was employed to investigate the temporal variation in the stock market. The study dataset consists of 2488 data points of logarithmic returns for each stock, which is one less than the number of the closing prices. The logarithmic return data was binned into a time window of size 500 data points (trading days) and a step-up of 20 data points. It resulted in 100 observations of length 500 data points, each separated by 20 data points corresponding to the dates from 1st June 2005 to 30th June 2015.

### Influence strength analysis

Analysis of weighted networks is performed at three levels: the complete network formed of 335 stocks; sector-specific, networks (15 sector specific networks); and industry-specific network (cement industry network as a special case).

**Fig 1:**
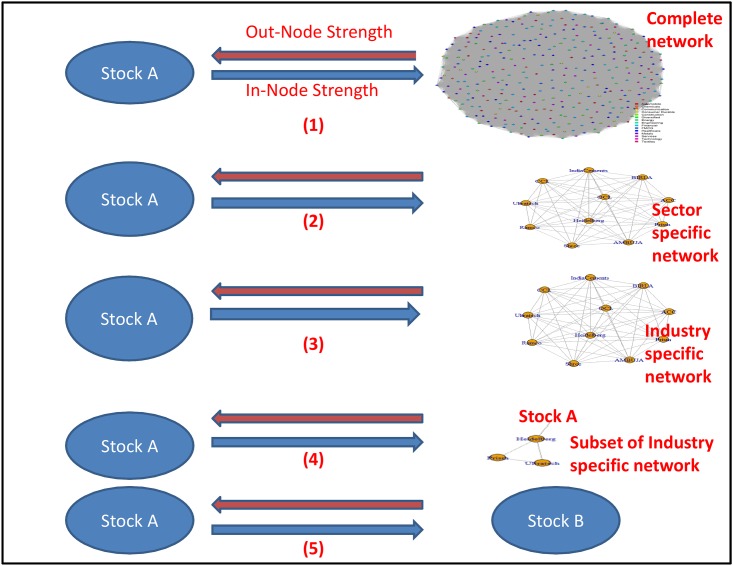
Influence Relationship model existent in an Equity network.

In the Fig 1, for the hierarchical levels 1, 2, 3, & 4; the blue arrows depict the influence of a given stock on an equity network (equity network may be formed in any of the following ways: network formed of selected 335 of S&P BSE 500; network formed by stocks belonging to a single sector; network formed by stocks belonging to a single industry; and network formed by a subset of stocks belonging to a given industry). The red lines for the hierarchical levels 1, 2, 3, & 4 depict the system-wide influence (here system represents the network) on a given stock. In hierarchical level 5, the blue line illustrates the influence of Stock A on Stock B; and the influence in the reverse direction is represented by the red line.

#### Influence strength computation methods

The influence strength measures are computed based on two approaches: (i) Cross-correlation based influence strength measure. We refer to this measure as cross-correlation based influence strength measure because of the fact that it measures the similarity of two series as a function of the time lag of one relative to the other. (ii) Market capitalization based influence strength measure.

Cross-Correlation based Influence strength measure: As discussed in the introduction section, the first attempt to compute a quantifiable measure for vertex strength of one stock in the network was done by Kim et. al. in the year 2002[37]. The step-wise computation method as proposed by Kim et. al. [37] is explained below. The following equation computes the log returns of the stock prices,

$$S_{i}=\text{ln}\left(\frac{CP_{t}}{CP_{t-1}}\right)$$ (1)

Where, *CP* is the closing price of a stock

The cross-correlations computed between individual stock’s logarithmic returns are represented in form of a matrix *C*. The elements of the matrix are computed using the equation below,

$$C_{i,j}\equiv \frac{\langle S_{i}S_{j}\rangle -\langle S_{i}\rangle \langle S_{j}\rangle }{\sqrt{\left(\langle S_{i}{}^{2}\rangle -{\langle S_{i}\rangle }^{2}\right)\left(\langle S_{j}{}^{2}\rangle -{\langle S_{j}\rangle }^{2}\right)}}$$ (2)

Where, <*S*_*i*_> refers to the average value of *S*_*i*_ for a given duration t

For the construction of a weighted network, two metrics are introduced: ‘residual log return’ *G*_*i*_*(t)* and ‘weighted cross-correlation coefficient’ *w*_*i*,*j*_. The first metric ‘residual log return’ represented by *G*_*i*_*(t)* captures the intrinsic properties of the changes in stock prices and is defined as follows:

$$G_{i}(t)=S_{i}(t)-\frac{1}{N}\sum_{i}S_{i}(t)$$ (3)

Where, *S*_*i*_*(t)* is the log returns of stocks as explained in Eq (1)), and *G*_*i*_ refers to the residual log return of a stock *i* after deducting the mean value of the log returns of a stock *i* for the number of data points (trading days) in a given observation.

The second metric ‘weighted cross-correlation coefficient’ represented by *w*_*i*,*j*_ is defined using the equation given below:

$$w_{i,j}\equiv \frac{\langle G_{i}G_{j}\rangle -\langle G_{i}\rangle \langle G_{j}\rangle }{\sqrt{\left(\langle G_{i}{}^{2}\rangle -{\langle G_{i}\rangle }^{2}\right)\left(\langle G_{j}{}^{2}\rangle -{\langle G_{j}\rangle }^{2}\right)}}$$ (4)

The weighted graph can be defined in the following manner, the vertices of the graph represent individual stocks, each pair (*i*,*j*) of vertices possess an edge between them. There is an associated weight *w*_*i*,*j*_ for each edge connecting two vertices. The total influence strength *s*_*i*_ of *i*^*th*^ stock can then be stated as a summation of the weights on the edges incident upon the vertex *i*. The influence strength of *i*^*th*^ stock in the time period *k* is defined by this method as,

$$s_{i}=\sum_{j\neq i}w_{i,j}$$ (5)

Market capitalization based Influence Strength: There are two key issues that need to deal while considering *w*_*i*,*j*_ as a sole measure of influence strength. The first issue that needs to be pointed out is that, are there any other contributing factors apart from cross-correlation coefficient that is ignored in the Eq 5? The second issue is that whether the influence of *i*^*th*^ stock on the *j*^*th*^ stock is the same as *j*^*th*^ stock on the *i*^*th*^ stock, i.e. whether the influence strength is symmetric or not. Taking these two issues into consideration, we introduce the second method proposed by Arora et. al. [38]. In this method, the computation of influence strength is done by including the values of market capitalizations of the interacting stocks.

This method computes the influence strength in two steps. According to this approach, the cross-correlation *w*_*i*,*j*_ values between *i* and *j* cannot be expressed as the measure of the influence of both *i* on *j* and *j* on *i*. This model is excessively oversimplified. According to the author [38], the real drawback of this model is that a stock with a high market capitalization and a stock with a small market capitalization are given equal importance in their contribution to their respective influence strengths. We elaborate this concept with an illustration mentioned below.

Suppose a raw material supplying company *i* with market capitalization *M*_*i*_(∼ INR 50,000 crores) is having a vertical linkage in the supply chain to a manufacturing company *j* with market capitalization *M*_*j*_ (∼INR 2,00,000 crores). And for example, the stock price of *i* is correlated to that of *j* with *wi*,*j* (∼ 0.6). In such scenarios, the current definition of influence would not persist as the magnitude of influence of stock *i* on *j* would be less than the influence of stock *j* on *i*. Because of this reasoning, the measure proposed by the Kim et al. [37] seems to be a true unfit for quantifying the influence strengths of stocks. Backed by this reasoning, the study by Arora et. al. [37] has proposed a new measure of influence strength which is given below,

$$\text{Infl}(\text{i}\to \text{j})={\text{w}}_{\text{i,j}}\left(\frac{{\text{M}}_{\text{i}}}{{\text{M}}_{\text{i}}+{\text{M}}_{\text{j}}}\right)$$ (6)

Where, *M*_*i*_ and *M*_*j*_ are the market capitalization of stock *i* & *j* in INR crores respectively. The influence strength of stock *i* in the network will be the sum of influences of *i* over all companies *j* in the network. Thereby, the market capitalization based influence strength will be computed using the following equation,

$${\text{s}}_{\text{i}}=\text{Infl}(\text{i}\to \text{j}).{\text{M}}_{\text{j}}=\sum {\text{w}}_{\text{i,j}}\left(\frac{{\text{M}}_{\text{i}}{\text{M}}_{\text{j}}}{{\text{M}}_{\text{i}}+{\text{M}}_{\text{j}}}\right)$$ (7)

#### Influence strength analysis for the complete weighted network

The weighted network is built as proposed by Kim et. al. [37,51]. For the construction of weighted networks the ‘weighted cross-correlation coefficient’ *w*_*i*,*j*_ is used (computation as in Eq 4). For each of the time-frame window (observation) as mentioned in the rolling window approach, weighted cross-correlation coefficients matrix between stocks was generated.

The log returns of the stock prices are computed using Eq 1. The weighted cross-correlations between individual stocks are represented in the form of a matrix C, and the elements of this matrix are computed by using Eq 4. This resulted in 100 observations of matrices of size 335 X 335, each of these matrix consisting of weighted cross-correlation coefficients values. Using this weighted cross-correlation matrices the weighted network is constructed. The complete weighted network is defined as follows: the vertices of the graph are represented by the 335 constituent stocks of BSE S&P 500 index. Each of the pair of vertices (*i*,*j*) has a connecting edge between them with an accompanying weight *w*_*i*,*j*_.

The influence strength is a metric to quantify the influence of a stock on the remaining stocks in the equity network. The value *w*_*i*,*j*_ (as in Eq 4) is the representative measure of the influence that the stocks exercise on each other. We employ two methods (cross-correlation based approach & market capitalization based approach) for the computation of the influence strength of the stocks constituting the complete weighted network and do a comparative analysis of listed scripts ranked based on their respective influence strength measure. For computation of the influence strength measure, the cross-correlation based approach uses Eq 5, and market capitalization based approach uses Eq 7. The weight *w*_*i*,*j*_ has a range between [−1, 1]; therefore some of the values of the influence strength in some particular nodes could be in negative form. For avoiding such situations, we consider the absolute value of influence strength for each vertex. We compute the influence strength measure of each of the 335 stocks for each of the 100 observations by utilizing both the cross-correlation based approach & market capitalization based approaches. The stocks are further ranked according to the descending order of their respective influence strength in both the approaches (cross-correlation based approach & market capitalization based approach). And, the rankings obtained are further compared with the factual economic situations currently existing in India. Based on the comparative analysis, the ranking provided by the ‘market capitalization based influence strength approach’ was found to be more meaningful than that of ‘cross-correlation based approach’. Henceforth, in the current study we only consider market capitalization based influence strength measure for the remaining investigative steps.

**Fig 2:**
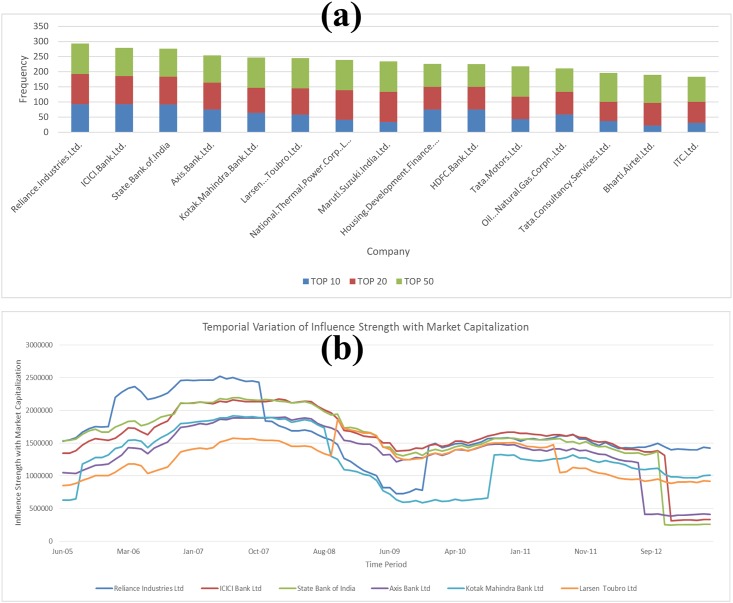
**(a)** Frequency of occurrence of top fifteen stocks in the top 10, 20 & 50 rankings in market capitalization based influence strength measures across 100 observations; (b) Temporal variation plot of market capitalization based influence strength of top six companies for 10 years period 2005–15.

We further explore the temporal variation of the market capitalization based influence strength for the top 6 ranked companies (on the basis of the average values of market capitalization based influence strength). Any single company cannot have a high influence over the market for a long period of time. For an investigation into the temporal variation of the high ranked companies over the past 10 years, we generate temporal variation plots (Fig 2(b)). There may be several reasons for the fluctuations of influence strength of the high ranked companies over the 10 periods. If any sudden highs and lows are noticed in some specific time periods, we attempt to explain it in terms of the historical happenings in macroeconomics changes in the domestic environment and international arena in those periods.

#### Sector wise influence strength analysis

We move our focus to sector specific weighted networks and their respective influence strength analysis. In agreement with the objective of sector specific network analysis, we segregate the 335 stocks according to their belongingness to one of the 15 sectors. The number of stocks belonging to each sector is given in Table 1. We generate 100 observations for each sector using rolling window approach (each observation with 500 data points and 20 data points’ step-up) and further generate weighted networks using the method explained before. We thereby obtain 100 weighted networks representing 100 temporal observations for each of the 15 sectors.

**Table 1:** Number of stocks in a given sector.

Name of the sector	Number of stocks in that sector(out of 335 stocks)
Automobiles	21
Chemicals	26
Communication	7
Consumer Durable	12
Construction	31
Diversified	10
Energy	20
Engineering	25
Financial	49
FMCG	34
Healthcare	34
Metals	18
Services	19
Technology	21
Textiles	9

For the constituent stocks of the 14 sector specific networks for 100 observations, market capitalization based influence strengths are computed. The stocks belonging to the diversified sector is excluded from the further analysis because all these stocks have revenues generated from diverse business segments and the influence relationships existent among these stocks is purely coincidental, and no macroeconomic reasoning can be attributed to it. The market capitalization based influence strength equation (original form is given in Eq 6) with minor constraints is used. The modified equation is as follows,

$${\text{s}}_{\text{i}}=\text{Infl}(\text{i}\to \text{j}).{\text{M}}_{\text{j}}=\sum {\text{w}}_{\text{i,j}}\left(\frac{{\text{M}}_{\text{i}}{\text{M}}_{\text{j}}}{{\text{M}}_{\text{i}}+{\text{M}}_{\text{j}}}\right)\;\text{i,j}\in \text{ Same sector}$$ (8)

Sector-wise, the stocks are further ranked to select the top 10 highly influential stocks. This step is done to identify the highest influential stocks of a given sector. Further, the average influence strengths of stocks belonging to constituent industrial segments of a particular sector are also computed and plotted. This step is required to identify the industry segment having the highest influence in a given sector. This information is relevant for financial analyst employed in portfolio management and equity research firms as it gives an indication of the industry they ought to track. We also investigate the temporal variation of mean influence strength of constituent industry segments of sectors across 100 observations. This will give a picture of the dominant industry segments in each sector and their influence relationship over their peers in the sectorial networks. The temporal patterns may reveal whether an industrial segment within a given sector is dominant throughout ten years period and whether there are radical variations (such sudden highs and lows) in its pattern.

#### Influence strength analysis & network analysis for a specific sectorial weighted network: a case of construction sector

We next go further deep into a specific sector network to understand its topology and influence relationships. We select the construction sector for a detailed study. We use the similar approach as employed for the complete weighted network to generate observation-wise weighted networks for the construction sector.

Influence strength analysis: We use the market capitalization based influence strength measure (original form is given in Eq 6) with minor constraints for computing the node strengths in the Construction sector network. The modified equation is as below,

$${\text{s}}_{\text{i}}=\text{Infl}(\text{i}\to \text{j}).{\text{M}}_{\text{j}}=\sum {\text{w}}_{\text{i,j}}\left(\frac{{\text{M}}_{\text{i}}{\text{M}}_{\text{j}}}{{\text{M}}_{\text{i}}+{\text{M}}_{\text{j}}}\right)\;\text{i,j}\in \text{ Construction sector}$$ (9)

The construction sector network consists of 32 stocks belonging to 7 different industrial segments.

Network Analysis: For understanding the statistical properties of the construction network, we investigate the small word characteristics and power law characteristics. For the purpose of deriving a reduced network that is free of noise and having a limited number of edges connected based on robust correlation structures, there is a necessity of performing network filtering steps. Several researchers have employed different network filtering methods for equity networks. Some of them are MST, Hierarchical Tree, Planar Maximally Filtered Graph, Random Matrix Theory based filtering and Threshold filtering. In the current study, we employ threshold filtering for deriving a filtered network of construction sector stocks. The findings from the study by Hu Ping and Xi Jie [52] demonstrated that in situations where the weighted correlation coefficient matrix is filtered by employing threshold approach, a large degree of fluctuations caused by correlations can be suppressed and subsequently a more stable topology can be derived.

For identifying a threshold level at which the network exhibit scale-free behaviour and power law distribution, first level optimization needs to be done. In this optimization technique, threshold filtered networks are generated for ranges of 0.05 to 0.75 (separated by 0.05 at each step-up). For a set of filtered networks (100 networks representing 100 observations) at a given threshold level, the mean number of degrees of the networks are computed. The mean number of degrees is plotted against the individual values of threshold range (0.05 to 0.75 separated by 0.05 at each step-up), and the point at which the maximum slope is obtained is noted. This forms our optimum threshold level. We investigate the scale-free characteristics and power law characteristics of filtered networks at this threshold level. We generate 100 construction networks of 32 stocks representing 100 temporal observations and filter them at the optimum threshold level. In this filtered networks, an edge between two nodes (stocks) exists if and only if the cross-correlation between the two nodes is higher than a specified threshold value θ. The adjacent matrix A for this network is represented by,

$${\text{A}}_{\text{i},\text{j}}=\{\begin{array}{ll} 1 & {\text{if w}}_{\text{i,j}}\;>=\theta \text{ and i}\neq \text{j}\\ 0 & \text{otherwise} \end{array}$$ (10)

This set of networks is referred to as empirical networks in our current study.

For investigating into the small word characteristics and power law characteristics of the construction sector network, there is a necessity of a comparable randomized network derived from the current empirical dataset. To fulfill this objective, the empirical log returns data of 32 construction industry stocks for the period 1^st^ June 2005 to 30^th^ June 2015 are shuffled 1000 times randomly in a row-wise manner to break the correlation structure among the stocks and to introduce randomness in the logarithmic return dataset. Post thousand times randomizations, each of the 32 stocks are checked for randomness using Run Test with a statistical significance of 0.05. Any stock failing the Run Test at this statistical significance is again randomized 1000 times and tested for statistical significance. This process is repeated till the complete dataset is randomized. Once this process is completed, rolling window approach is used to generate 100 observations of length 500 data points and step-up size of 20 data points. These randomized observations are used to produce correlation matrices and networks. Because the log returns data is totally randomized, the networks derived from this dataset will also be random. Using this dataset, we generate 100 networks of 32 stocks representing 100 temporal observations and filter them at optimum thresholds obtained in the earlier step. This set of networks is referred to as random networks in our current study.

Scale free behaviour: For investigating the small-world characteristics of the construction network, we introduce two network metrics, average path length, and average clustering coefficient.

If the empirical network has a smaller average path length and a larger average clustering coefficient in comparison to random networks at all observations, it is assumed that the empirical network follows small-world characteristics [53]. The two conditions for small world characteristics are: (i) average path length of the empirical network should be less than of random network; (ii) average clustering coefficient of the empirical network should be more than that of a random network. The average path length and average clustering coefficient are computed as mentioned in the study by Li Zhua et. al. [54].

The average clustering coefficient and average path length for 100 filtered empirical networks & 100 random networks (each network represents one observation generated using rolling window approach) are computed and are further plotted.

Power law characteristics: The power law characteristics are investigated for the set of empirical networks filtered at 0.25 thresholds. The probability of occurrence of degree k in a given vertex is given by,

$$\text{P}(\text{k})\;\alpha \;{\text{k}}^{-\gamma }$$ (11)

or, equivalently,

logP(k) α -γlogk (12)

The above equation indicates that one can plot this relationship as a straight line. We compute the power law exponents and inverse power law exponents of the filtered empirical networks at 0.25 threshold levels for 100 observations.

#### Influence strength analysis & network filtering for an industry specific network: a case of cement industry

Influence Strength Analysis: We next move our focus to industry specific weighted network analysis. We select the cement industry, an important one in the construction sector, the cement industry for our further study. Out of the 32 stocks constituting the construction sector network, 11 stocks belong to the cement industry. We compute the market capitalization based influence strength of each of the cement stocks inside the cement industry network by following the earlier approach and a modified equation given by,

$${\text{s}}_{\text{i}}=\text{Infl}(\text{i}\to \text{j}).{\text{M}}_{\text{j}}=\sum {\text{w}}_{\text{i,j}}\left(\frac{{\text{M}}_{\text{i}}{\text{M}}_{\text{j}}}{{\text{M}}_{\text{i}}+{\text{M}}_{\text{j}}}\right)\;\text{i,j}\in \text{ Cement Industry}$$ (13)

Network Filtering: In the market capitalization based influence strength measure, two major contributors are market capitalization in INR and correlation structure as a matrix form. The magnitude of market capitalization dependent on the price of stock on a trading day and volume of stocks currently traded in the market. In addition to this, the stock splits and dividends also have an impact on the market capitalization value. Since the prices of stocks are highly volatile and non-linear in nature, the market capitalization also has an inherent volatility feature in it. The correlation structure which is the contributing component is comparatively more stable as it is dependent on common industry specific forces affecting a group of related stocks. In this section, we further filter the cement industry network by employing two different filtering approaches; and using these filtered networks in the next section, we attempt to build an econometric model based on industry specific macroeconomic measures.

For the purpose of deriving a reduced network that is free of noise and having a limited number of edges connected based on robust correlation structures, there is a necessity of performing network filtering steps. We employ the dual approach of threshold filtering and MST for generating filtered networks of cement industry stocks.

Threshold filtering method: The first approach used is threshold filtering. We follow the threshold filtering methodology as discussed in the construction network analysis in the above sections. The mean number of degrees is plotted against the range of values of thresholds (0.2 to 0.45 separated by 0.05 at each step-up), and the point at which maximum slope is obtained is noted. This point is referred to as optimum threshold level. We generate 100 weighted networks representing 100 temporal observations and filter them at this optimum threshold level. We visually inspect those filtered networks for 100 observations to identify consistent sub-graph formation.

MST Analysis: The second noise filtering technique used is Minimum Spanning Tree(MST). The MST is a type of spanning tree having the shortest length of all spanning trees and is derived using Prim’s algorithm. It is a kind of acyclic graph with all nodes connected via edges. For the purpose of building the MST we employ distance metric equation proposed by R.N. Mantegna et al. [25].

$$d_{i,j}=\sqrt{2(1-p_{i,j}})\text{ 0}\leq d_{i,j}\leq 2$$ (14)

Where, *p*_*i*,*j*_ refers to the correlation between log returns of stocks *i* and *j*. Here *p*_*i*,*j*_ *= C*_*i*,*j*_ (from Eq 2). The distance among any two stocks *i* and *j* is referred to as *d*_*i*,*j*_ and is called as subdominant ultrametric distance. Using the values of *d*_*i*,*j*_, we generate 100 Distance Matrices for 100 observations and derive the MST plots observation-wise. We visual inspect those MSTs for 100 observations to identify consistent sub-graph formation.

## Results and Discussion

### Influence strength analysis results of the complete network

The list of stocks ranked in descending order according to their mean influence strength and frequency of occurrence of stocks in top 10 positions across 100 observations in the cross-correlation based influence measure is depicted in Table 2. A similar list derived using market capitalization based approach having the frequency of occurrence of stocks in top 5 and top 10 positions across 100 observations is depicted in Table 3.

**Table 2:** List of stocks with highest top 10 mean influence strength based on cross-correlation coefficients.

Rank	Company Name	Frequency of occurrence in top 10
1	IDBI Ltd	99
2	Century Textiles and Ind Ltd	92
3	Reliance Capital Ltd	80
4	Vijaya Bank	73
5	Dena Bank	66
6	UCO Bank	35
7	Reliance Infrastructure Ltd	49
8	IFCI Ltd	44
9	Syndicate Bank	16
10	Andhra Bank	24

**Table 3:** List of top 10 ranked stocks with highest frequency in the top 10 rankings based on market capitalization based influence strength across 100 observations.

Rank	Company	Frequency of occurrence in top 10 rankings	Frequency of occurrence in top 20 rankings	Frequency of occurrence in top 50 rankings
1	Reliance Industries Ltd.	93	100	100
2	ICICI Bank Ltd.	93	93	93
3	State Bank of India	92	92	92
4	Axis Bank Ltd.	75	89	90
5	Kotak Mahindra Bank Ltd.	64	83	100
6	Larsen and Toubro Ltd.	58	87	100
7	National Thermal Power Corporation Ltd.	41	98	100
8	Maruti Suzuki India Ltd.	34	100	100
9	Housing Development & Financial Corporation Ltd.	75	75	76
10	HDFC Bank Ltd.	75	75	75

The influential stocks ranked according to the mean influence strength for 100 observations were IDBI Bank, Century Textiles & Industries Ltd, Reliance Capital, Vijaya Bank, Dena Bank and UCO Bank. It was quite a surprise to observe that light weighted stocks having a limited impact on the Indian economy & industrial growth are ranked at top positions. On the other hand, the heavyweight stocks such as Reliance Industries, ICICI Bank Ltd., State Bank of India, Axis Bank Ltd., Kotak Mahindra Bank Ltd. and Larsen & Toubro Ltd. are all landed at low rankings with low mean influence strength. For investigating the reason behind the light weight stocks listing up in top positions in the current calculations, we estimate the mean absolute correlation coefficient of these stocks with the remaining 334 stocks in the network. Upon careful examination of the mean absolute correlation coefficient figures, we can notice that all these light weight stocks such as, IDBI Bank, Century Textiles & Industries Ltd., Reliance Capital, Vijaya Bank and Dena Bank have pretty high levels of correlation figures ranging between 0.334 and 0.346; whereas, the heavyweight stocks have comparatively lower levels of correlation values ranging between 0.259 and 0.292. It is to be noted that the terms, ‘heavy weight’ and ‘light weight’ are mentioned in reference to the stock’s weight in the S&P BSE 500 index calculations.

The stocks selected based on rankings of market capitalization based influence strength provides us with a relatively better acceptable rankings to the companies. It can be observed in Table 3, that Reliance Industries, ICICI Bank, and SBI have appeared 92 times in top 10 positions across 100 observations. This reflects their consistent influence on the Indian equity market for the study period 1^st^ June 2005 -30^th^ June 2015. Out of these three companies, the first one is the largest industrial conglomerate in India, the second being the largest private bank in India and the third being the largest nationalized bank in India. In the rankings table, Reliance Industries have been consistently ranked at the first place in top 5 and top 10 rankings. The ranking of the Reliance Industries at first position is definitely relevant in economic sense, since as on May 2015, Reliance Industries is reported to be the second most profitable company in India [55]; and as on March 2013 it is the second-largest publicly traded company in India equity market in terms of market capitalization [56]; and as on August 2013 it is reported to be the second-largest company in the country in terms of its revenues [57]. In the year 2015, the Fortune 500 has listed Reliance Industries Ltd. at 158^th^ position in its annual rankings [58]. As per the Annual Reports 2012–13, Reliance Industries Ltd. has contributed to nearly twenty percent of India's total exports [59].

The second highly influential stock in the current analysis is ICICI Bank. ICICI Bank is the largest private sector banking organization in India and as on March 31, 2015, it had total assets of Rs. 6,461.29 billion (US$ 103 billion) and has generated profit after tax of Rs. 111.75 billion (US$ 1,788 million) for the Financial Year 2014–15 [60]. A wide variety of banking products and financial services are offered by ICICI Bank to its corporate and retail customers. It has specialised subsidiaries in the domains of venture capital investments, investment banking, life insurance, non-life insurance, and asset management.

The third highly influential stock in the current analysis is the SBI. It is the largest banking and financial services company in terms of the yearly turnover and total assets (as on Dec 2015). State Bank of India is a Indian public sector bank having its presence in many countries globally. The key products and revenue segments of SBI include income from investment, interest & discount on advances & bills, interest on balances with RBI and other inter-bank funds [57, 58].

One may point out that the stocks are ranked high in the market capitalization based approach because of their large market capitalization values. In this regard, we may clear the doubt concerning the undue importance of market capitalization by doing a comparative study of two companies (SBI and Housing Development and Finance Corporation Ltd.) possessing similar levels of market capitalization but with a huge difference in rankings in influence table. In this comparative analysis, we notice that despite of SBI and Housing Development Finance Corporation Ltd having similar levels of market capitalization (SBI has market capitalization of INR 202320.4 crores and Housing Development Finance Corporation Ltd. has market capitalization of INR 203361.81 crores as on 8^th^ July 2015), SBI is ranked at the 3^rd^ position and the Housing Development and Finance Corporation Ltd. is ranked at 9^th^ position in Table 3.

The frequency of occurrence of top fifteen stocks in the top 10, 20 & 50 rankings according to the market capitalization based influence strength across 100 observations is plotted in Fig 2(a). The temporal variation in the market capitalization based influence strength for the top 6 stocks over their ten years period (June 2005- June 2015) is given in Fig 2(b). We can observe in this plot that Reliance Industries has a dominant role in market capitalization based influence during the period June 2005-October 2007. Abruptly, there is a noticeable dip in the influence strength of Reliance Industries just before the sub-prime financial crisis in September 2008. In the post-December 2009 era, the influence of Reliance Industries has risen abruptly, and subsequently it maintained a dominant position till December 2015. We can also observe that ICICI Bank, SBI and Axis Bank have similar levels of co-movements in their strengths over ten years period. SBI has been dominating among these three banks for the period June 2005 to June 2009. Post that phase, ICICI has maintained a slight edge over SBI especially during the period June 2009 to 2015. Axis Bank has consistency maintained a subordinate position to SBI and ICICI over the past ten year’s period. We can also observe that Kotak Mahindra Bank had a co-movement with SBI, ICICI Bank, and Axis Bank during the period Sep 2005 till Aug 2008, the time when the financial crisis started. Post that period; it has maintained a very weak position in comparison to the other three banks till Jan 2011, after that one can notice co-movement of Kotak Mahindra Bank with the three other major banks.

During October 2007 to April 2010, there was a change in the international environment as the sub-prime financial crisis originating in the US has spread its effect globally and this has resulted in a reduction of credit access for industrial growth leading to the dampening of the exports scenario globally. During this period, we can notice that the influence of Reliance Industries over the equity market has declined. SBI, ICICI Bank and Axis Bank had very less exposure to the financial crisis as they were not involved in complex financial engineering instruments and could withstand the shock. In early 2015, we can notice a sudden dip in the influence of stocks belonging to SBI, ICICI and Axis Bank. This is because over a period these banks have accumulated a large chunk of Non-Performing Assets and the subsequent regulatory tightening by RBI have impacted the investor sentiment on these banks resulting in lower valuations. ICICI Bank currently has accumulated the highest levels of NPAs in the big private banking sector in India and the Gross NPA as on January 2016 is estimated to be 3.8 percent mark for the last three-quarters. SBI has accumulated high levels of NPAs and as on January 2016, it is reported to be at 4.2 percent (gross).In addition to this, SBI also has exposure to different industrial sectors which are currently under stress. Axis Bank also has the second largest NPA in the private banking space, after ICICI Bank [61].

We surprisingly notice that Kotak Mahindra Bank does not have a similar dip in influence compared to the other three banks. This is due to the fact that the revenue model of Kotak Mahindra Bank has been quite different from these three other banks, as it focussed more on leasing and financial businesses rather than retail operations. The other important stock in the influence ranking list is L& T Ltd, the largest capital goods manufacturer in the country.

### Sector wise influences analysis results

The highest influential stock of each sector based on the mean influence strength across 100 observations is given in Table 4.

**Table 4:** Sector wise highest influential stocks and their respective industry segments.

Sl. No.	Sector	Highly influential stock	Industry Segment the stock belong
1	Automobiles	Tata Motors Ltd.	Cars & Multi Utility vehicles
2	Chemicals	Tata Chemicals Ltd.	Soda Ash
3	Communication	Tata Communications Ltd	Telecom Services
4	Consumer durables	Voltas Ltd.	AC & Refrigerators
5	Energy	Reliance Industries Ltd.	Crude Oil and Natural Gas
6	Engineering	Siemens Ltd.	Switching Equipment
7	Finance	ICICI Bank Ltd.	Banking
8	FMCG	Hindustan Unilever Ltd.	Cosmetics & Toiletries
9	Healthcare	Cipla Ltd.	Drugs & Pharma
10	Metals	Tata Steel Ltd.	Finished Steel
11	Services	Zee Entertainment Enterprises Ltd.	Recreational services
12	Technology	Tata Consultancy Services Ltd.	Compute Software
13	Textiles	SRF Ltd.	Synthetic Fabrics
14	Construction	Ultratech Cements Ltd.	Cement

The highest influential stock of a given sector may or may not belong to the highest influential industry segment in that sector. The five most influential stocks based on mean influence strength of each of the 14 sectors is plotted and is exhibited in the S4–S6 Figs. For each of the sectors, the mean influence strength of stocks belonging to the industrial segments constituting the given sector is also computed and plotted. The highest influential industrial segment in each of the 14 sectors is given in Table 5.

**Table 5:** Sector wise highest influential industry segments.

Sl. No.	Sector	Highly influential industrial segment
1	Automobiles	Cars & Multi Utility Vehicles
2	Chemicals	Soda Ash
3	Communication	Telecom Services
4	Consumer durables	Consumer Electronics
5	Energy	Crude Oil & Natural Gas
6	Engineering	Switching Equipment
7	Finance	Banking
8	FMCG	Cosmetics & Toiletries
9	Healthcare	Drugs & Pharma
10	Metals	Others Non-Ferrous Metals
11	Services	Most industry segments have uniform influence
12	Technology	Computer Software
13	Textiles	Synthetic Fabrics
14	Construction	Cement

The mean influence strength analysis plot of all the industry segments constituting each of the 14 sectors is given in S1–S3 Figs. This will provide insights into the relatively important industrial segments in each sector which can largely affect the movement of constituting stocks of that sector.

**Fig 3:**
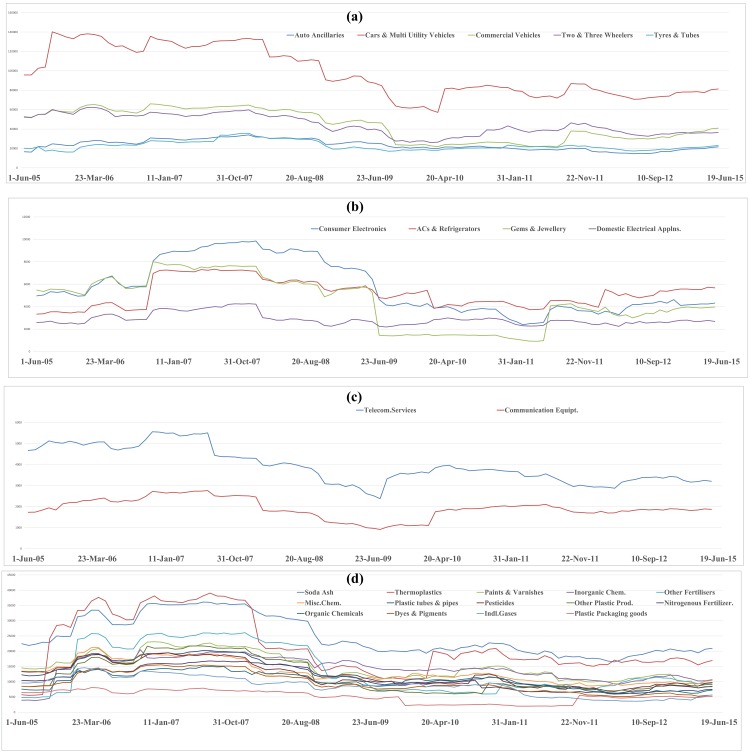
Temporal variation plot of mean influence strength of stocks belonging to the Automobiles sector, Consumer Durables sector, Communication sector and Chemicals sector.

**Fig 4:**
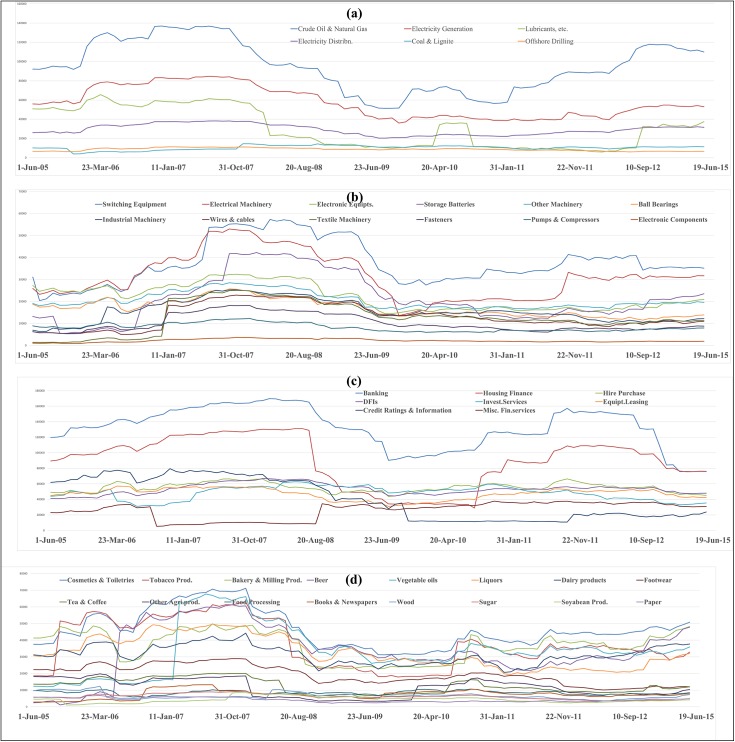
Temporal variation plot of mean influence strength of stocks belonging to the Energy sector, Engineering sector, Finance sector, and FMCG sector.

**Fig 5:**
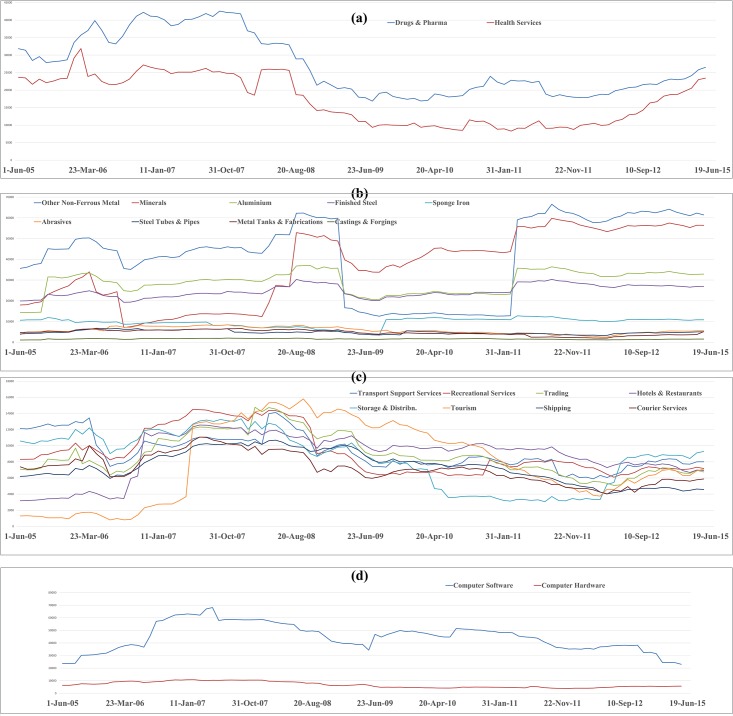
Temporal variation plot of mean influence strength of stocks belonging to the Healthcare sector, Metals sector, Services sector and Technology sector.

**Fig 6:**
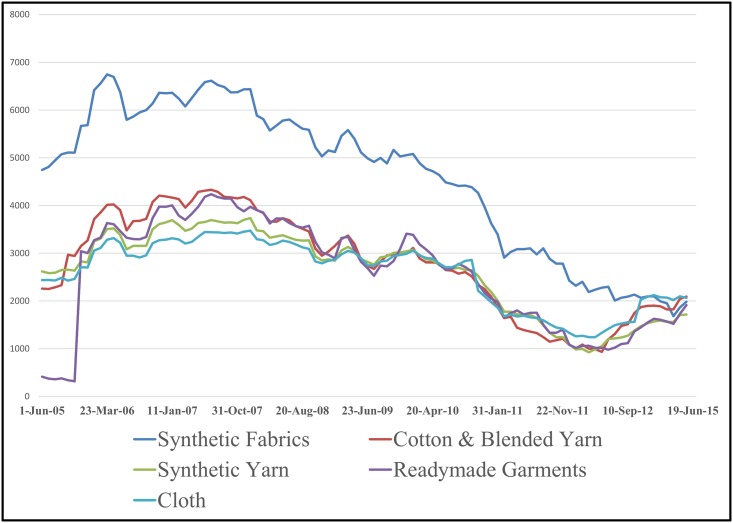
Temporal variation plot of mean influence strength of stocks belonging to the Textiles sector.

The temporal variation plot of mean influence strength of different industry segments constituting given sectors for ten years duration is depicted in the Figs 3–6. The temporal plot gives insights whether an industrial segment within a sector is consistently influential or are there any sudden variations in the temporal movement. We next discuss some of the key findings from the influence strength analysis of the 14 sectorial networks.

In the Automobile sector, we can observe in both, segment wise influence strength analysis plot (S1 Fig) & temporal variation plot (Fig 3(a)), that stocks belonging to the Cars and Multi-Utility Vehicles segment have the highest influence over the network. In temporal variation plot, we can also observe that stocks belonging to Tyres and Tubes segment and Auto ancillaries segment have similar levels of co-movement across ten years period. This is evident from the fact that the automobile sector is a vertically integrated sector, and the demand of Tyres and Tubes & Auto Ancillaries are highly linked to the growth of the Cars & Multi-Vehicle segment, Commercial Vehicle segment, and Two & Three wheelers segment respectively.

In the Chemical sector, we can observe in the segment wise influence strength analysis plot (S1 Fig) that stocks belonging to the soda ash industry have the highest influence on the remaining stocks in the chemicals sector network. Soda ash is an important component for manufacturing of glass, soaps, and detergents. The Indian glass industry is highly influenced by the cost variations in the soda ash. In the temporal variation plot of the chemical sector (Fig 3(b)), we can observe that stocks belonging to Thermoplastics segment have dominated in the initial phases of the study period. Close to the 2008 financial crisis, the influence of Thermoplastics segment has suddenly declined and henceforward it has remained low. There is a clear domination of stocks belonging to soda industry segment for a long period of time.

In both, the segment wise influence strength analysis plot (S1 Fig) & temporal variation plot (Fig 3(c)), of the communication sector, we observed that stocks belonging to the Telecom services industry have an edge on that of Communication Equipment industry. This trend is obvious since the growth of landline subscribers, mobile subscribers, and Internet subscribers dictate the growth in demand for telecommunication infrastructure on the ground.

In the influence strength analysis plot (S1 Fig) of consumer durables sector, we can observe that stocks belonging to the consumer electronics industry have an upper edge in influencing the network. The consumer durables stocks are highly influenced by the growth in the economy and increase in the disposable income leading to increased demand for consumer durables in urban, semi-urban and rural areas of the country. In the temporal variation plot (Fig 3(d)) of the consumer durables sector, we can observe that there is a stiff competition between stocks belonging to Consumer Electronics segment, AC & Refrigerator segment and Gems & Jewellery segment. There is no single leader for the entire ten years duration.

The majority of the stocks in the energy sector belong to the petrochemical based industries, which rely heavily on foreign exports of crude oil and its prices in the international market. In the both segment wise influence strength analysis plot (S2 Fig) & temporal variation plot (Fig 4(a)) of the energy sector, we can observe that stocks belonging to crude oil & natural gas producing companies occupy the highest position in the mean influence strength. This is followed by stocks belonging to Electricity generation companies. In India, the electricity generation is mostly dependent on thermal sources generated from coal, followed by gas based power stations, nuclear, hydroelectric and wind energy. In this context, the business performance of crude oil & natural gas producing companies can affect their stock price movement, which can subsequently influence the movement of stocks of large crude oil users (such as, electricity generation companies, lubricants producing companies) and offshore drilling companies. So, we can notice from the findings that the energy sector is a vertically integrated sector, and hence the influence is channelized among the different players of the vertical integration chain. In the temporal variation plot, we can also notice electricity generation segment has a perfect co-movement with the Crude Oil and Natural Gas industry segment.

In the engineering sector, we can observe that the Switching Equipment Manufacturing segment has the highest average influence strength across 100 observations. In the temporal evolution plot (Fig 4(b)) of engineering sector, we can notice that stocks belonging to switching equipment manufacturers and electrical machinery manufacturing companies are having dominant influence strength across 100 observations.

In both the segment wise influence strength analysis plot (S2 Fig) & temporal variation plot (Fig 4(c)) of the finance sector, we can observe that the stocks belonging to the banking sector possess the highest dominance in the network. In the temporal variation plot, we can also observe a perfect co-movement of stocks of the banking sector and the housing finance sector.

In the industry segment wise influence strength analysis plot (S2 Fig) of FMCG sector we can observe that stocks belonging to ‘Cosmetics & Toiletries’ segment occupy the highest position. The temporal variation plot (Fig 4(d)) of the FMCG industry is very dense, and we see intense competition among different industrial segments for domination. For an extensive period of time, stocks belonging to ‘Cosmetics & Toiletries’ segment have dominated the network. Apart from this segment, there seems to be a tough competition among Tobacco producers, Vegetable Oil producers, Beer producers, Bakery & Milling Products companies.

In the Healthcare sector, especially in pharmaceutical industry, there may not be any direct macroeconomic linkage leading to influence relationships among firms. This is because each pharmaceutical company has its portfolio of drugs, biological, diagnostics kits and the growths of each of these market segments finally affect their valuation & price movement. Because an equity market behaves as a complex system, the interactive patterns generated by such networks may or may not have economic rationality but may be a pure outcome of investing behaviours. We can observe that stocks belonging to companies engaged in Drugs & Pharma business have an edge in both industry segment wise influence strength analysis plot (S3 Fig) & temporal variation plot (Fig 5(a)).

In the industry specific analysis plot (S3 Fig) of the metals sector, we can observe that stocks belonging to companies involved in ‘Other Non-Ferous Metals’ business are having the highest mean influence strength over ten years period. In the temporal variation plot (Fig 5(b)) of the Metals sector, we can observe that stocks belonging to Other Non-Ferrous Metal segment have a continous domination over the network. However, we can observe that during August 2008 –Jan 2011, there is a sudden dip in the influence of the OtherNon-Ferrous Metal stocks. Post that period, the Other Non-Ferrous Metal stocks are highly influential. The Minerals processing industrial segment and Aluminium processing industrial segment also exhibit high levels of influence over the network for a long period of time.

The Services sector is a highly diversified sector consisting of entertainment industries, hotel chains, Logistics companies, Shipping companies, Travel & Tourism service providers, Retail chains and other services. In this context, the influence of one stock on another stock is purely based on behavioural investing and not on macroeconomic linkages. In the segment wise influence strength analysis plot (S3 Fig), we can observe that the stocks belonging to the Transport support companies have the highest influence in the network. In the temporal variation plot (Fig 5(c)) of the services sector, we can observe that no single industry segment has a consistent dominance over a long period of time. This is evident from the fact that in the services sector, each industrial segment is different and there is no macroeconomic linkage between those segments; therefore the influence of stocks on one another is purely coincidental and an outcome of behaviorial investing.

The Technology sector stocks either belong to software companies or computer hardware companies. In the software industry, most of the Indian IT companies listed in BSE are IT service providers and have their major chunk of their revenues generated from outsourced projects obtained from international markets. In such circumstances, international macroeconomic factors such as the change in exchange rates, business growth in US markets, UK Markets, European markets, economic conditions in Europe play a major role in the profitability of these companies and subsequently the movement of their stock prices. In both the segment wise influence strength analysis plot (S3 Fig) & temporal variation plot (Fig 2(d)) of the technology sector, we can observe that stocks belonging to the Computer software industry are clearly dominant.

The textiles sector is vertically integrated sector, and multiple macroeconomic factors may affect the valuation of the stocks giving rise to an influence relationship framework. Some of the macroeconomic factors that may affect the textiles sector are, the cost of cotton in domestic & international markets, the level of production of cotton in India, level of production of cotton globally, the demand for garments in domestic and international market, and the competitive position of India among the other major textile exporting countries like China, Bangladesh, Italy, Germany, Turkey and Vietnam. In both, the segment wise influence strength analysis plot (S3 Fig) & the temporal variation plot (Fig 6) of Textile sector, we can observe that stocks belonging to the companies involved in Synthetic fabrics business have clear dominance over the entire network.

We have not conducted sector specific network analysis for stocks belonging to the diversified sector (as classified by BSE) as it will not give any meaningful insights. This is because the companies listed under the ‘diversified category’ in BSE has revenues coming from diversified set of business segments and stock price correlations among those companies will not have any rationality.

### Influence strength analysis & network analysis results of the construction sector

**Fig 7:**
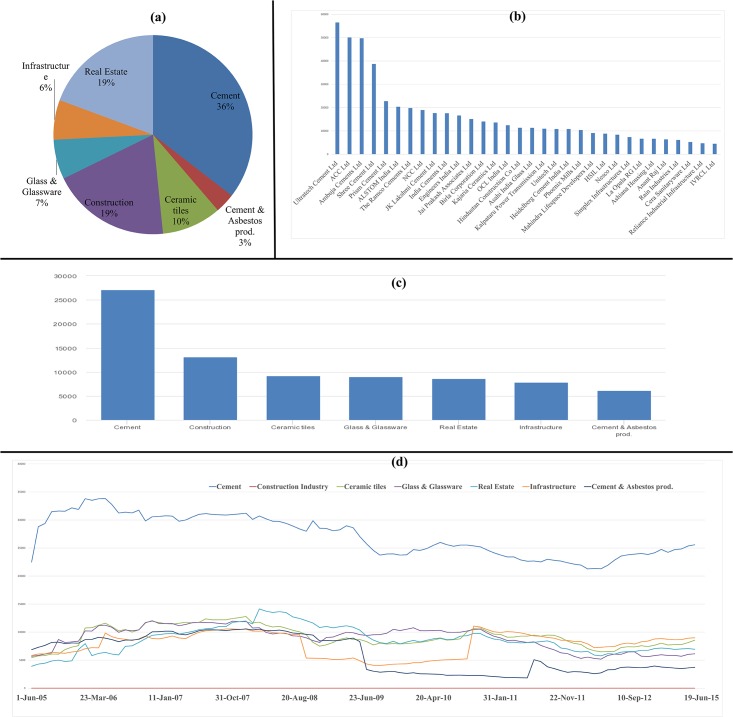
(a) The percentage distribution of construction stocks in different constituting industry segments; (b) The mean influence strength of the stocks belonging to construction sector across 100 observations; (c) Mean influence strength of each industry segment within construction sector across 100 observations; (d) Temporal variation of mean influence strength of constituting industry segments of construction sector for 10 years period 2005–15.

The percentage distribution of construction stocks in different constituting industry segments is depicted in Fig 7(a). The mean influence strength of the stocks belonging to construction sector across 100 observations is plotted in Fig 7(b). In both the industry specific analysis plot (Fig 7(c)) & temporal variation plot (Fig 7(d)) of the construction sector, we can observe that stocks belonging to cement industry are the highly influential.

**Fig 8:**
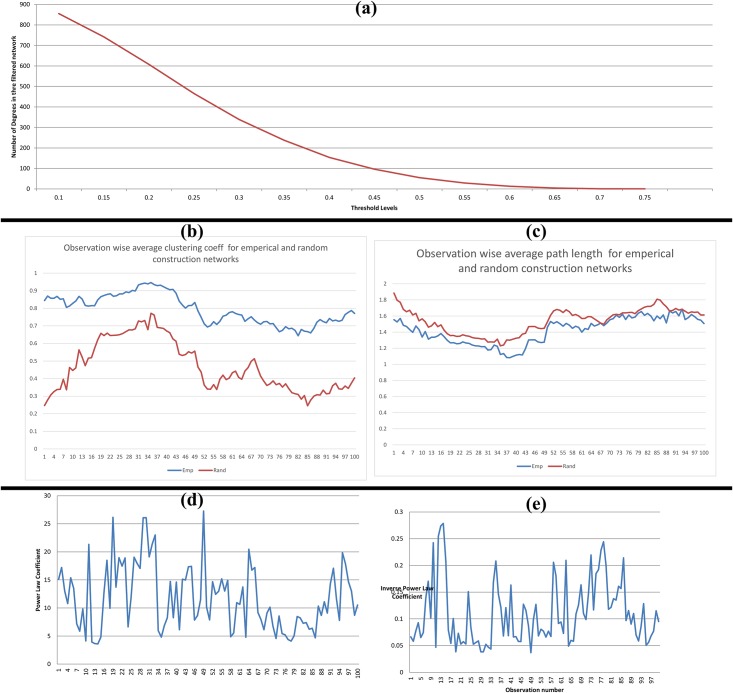
(a) Plot of mean number of degrees at different threshold levels against the threshold levels; (b) Plot of average clustering coefficient of empirical network & random networks across 100 observations; (c) Plot of average path length of empirical network & random networks across 100 observations;(d) Plot of power law coefficient for empirical networks across 100 observations; (e) Plot of inverse power law coefficient for empirical networks across 100 observations.

The plot of the mean number of degrees at different threshold levels against the threshold levels is displayed in Fig 8(a). The threshold level at which the slope is maximum is derived using interpolation and is noted to be 0.25.

As explained in the methodology, the scale-free characteristics and power law characteristics are investigated for the filtered networks at threshold level 0.25.

On analysing the filtered weighted network at threshold level 0.25, we can notice that the average path length of 100 observations for the empirical construction sector network is 1.421, and that of such random network is 1.540. In Fig 8(c), we can note that throughout the 99 observations (out of 100 observations), we see the average path length of the empirical networks is distantly less than that of random networks.

The average clustering coefficient for the empirical construction sector network across 100 observations is 0.794, and that of such random network is 0.467. In Fig 8(b), we can note that throughout the 100 observations, we see the average clustering coefficient of empirical network distantly greater than that of random networks. As the construction sector fulfils the two axioms of small world effect, we can hence refer the construction network as small world network.

We can observe in the plot of power law coefficients (Fig 8(d)) and inverse power law coefficients (Fig 8(e)) for 100 observations that the values are former is consistently high and later is consistently low across 100 observations. The average power law coefficient and inverse power law coefficients for 100 observations are obtained to be 11.7917 and 0.110378 respectively. If the magnitude of γ is large, there would be the presence of a lesser number of nodes possessing a large number of connections in the network. This indicates the presence of hub nodes within the construction sector network. One can interpret the inverse value of γ as a measure of the inclination of a node to form a connection with another node. If the value γ decreases, subsequently the magnitude of its inverse form γ^-1^ will increase. This indicates the predisposition of the network to have an increase in the number of nodes with high levels of interconnections.

### Influence strength analysis & network filtering results of the cement industry network

**Fig 9:**
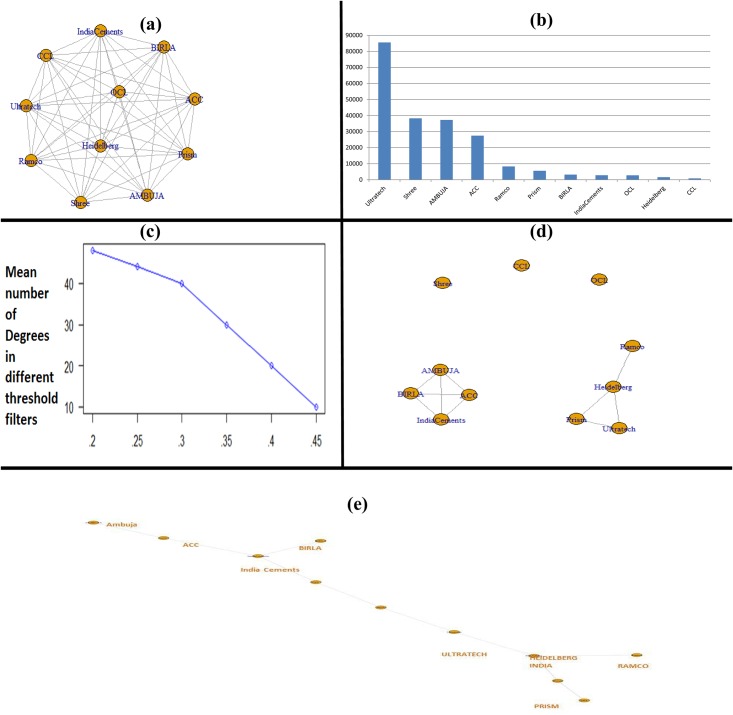
(a) A representative weighted network of cement stocks, formed by 11 cement companies; (b) Plot of mean market capitalization based influence strength for the 11 cement companies; (c) The plot of mean number of degrees at different threshold levels; (d) A representative filtered graph at 0.3 threshold level; (e) A representative Minimum Spanning Tree for the cement stocks for specific observation.

A representative weighted network of cement stocks, formed by 11 cement companies is given in Fig 9(a). The mean market capitalization based influence strength for the 11 cement companies is plotted in Fig 9(b).

The plot of the mean number of degrees at different threshold levels against the threshold levels is displayed in Fig 9(c). The threshold level at which the slope is maximum is derived using interpolation and is noted to be 0.3.

On visual inspection of the 100 threshold filtered networks (representing 100 synchronous temporal observations), we can clearly notice the formation of two prominently visible independent sub-graphs over an extended period of time. A representative filtered graph at 0.3 threshold level is shown in Fig 9(d).

On visual inspection of the 100 MST plots (representing 100 synchronous temporal observations), we can notice two distinct sub-graph formations analogous to the ones observed in filtered networks at 0.3 thresholds. A representative Minimum Spanning Tree for the cement stocks for specific observation is given in Fig 9(e).

### Rationale behind a stock being highly influential

We have observed the influential relationship models of global equity network, sector-specific networks, and industry specific networks. In these three hierarchies of influence relationships, we have observed that some stocks have consistently been highly influential through an extensive period of the time-line. We attempt to do some insightful analysis to reveal the reasons behind such a dominant influence over long periods of time.

#### Highly influential in global equity network

The highly influential stocks in the global equity network consist of a highly diversified company (Reliance Industries Ltd.) and remaining top five influential stocks consists of Banks.

Coming to the high influence of Reliance Industries Ltd, we can observe that Reliance Industries Ltd. is the major company under Reliance Group which has a lot of other subsidiary and associate companies that are listed in the Bombay Stock Exchange. Some of the important subsidiaries and associate companies are Reliance Retail, Reliance Life Sciences, Reliance Institute of Life Sciences, Reliance Logistics, Reliance Clinical Research Services, Reliance Solar, Relicord, Reliance Jio Infocomm Limited, Reliance Industries Infrastructure Ltd and a major mass media company Network18. On observing the business portfolios of Reliance Industries Ltd. and its subsidiaries and associate companies, it is clear that this group is highly diversified and has its presence in almost major business activities that are vibrant in a developing economy like India. Hence, its influence on the market is very significant. We can also observe in BSE that performance of one script of Reliance group affects the performance of other scripts of the Reliance group, even though there may not be any fundamental linkage between the firms. To explain this association, one must look into the shareholding pattern of the constituent stocks of BSE S&P 500. On careful examination of the shareholder pattern of the constituent stocks of BSE S&P 500, one can observe that biggest chunk is still held by the promoter group, which is 51 percent of the total market value as on Dec 2015. This means that only 49 percent of the equity is marketable. This is the reason behind the existence of influence relationships in stocks belonging to same promoter group. So a rise and fall of Reliance Industries Ltd. give an indicative perception in the market resulting in other stocks of Reliance moving in tandem with Reliance Industries Ltd.

Now coming to high influential role of the banking stocks, it is quite evident that in an emerging economy like India, banking has the prime, foremost and most significant share in shaping up the economy and influencing the other sectors. Especially in an Indian context, banks play a mix of key roles which includes, mobilizing savings through the network of branch banking that contributes to the formation of capital, which subsequently form the foundation of economic development activities; financing the industrial sector; promotion of entrepreneurial activities by underwriting the shares of new and existing companies [62]. So, it is natural for stocks of banking organizations to be ranked high.

All these influential stocks share some common characteristics such as, their exposure to all the major pillars of the economy, sensitivity to changes in international market (such as currency fluctuations, crude oil prices fluctuation, global demand for goods and services, economic growth in major trading countries, geopolitical tensions), highly integrated with domestic and global supply chain, dependency on the performance of Indian economy. So, as we can notice, the factors common to these companies also have a large impact on the other companies constituting the S&P 500 Index, thereby its movement plays a highly influential role in the movement of other stocks. In addition to this, most of these companies are heavy-weighted scripts in S&P BSE SENSEX, NSE NIFTY which are the barometers of the Indian equity market and in a broad sense the Indian economy. The general direction that these indices as whole take in short-run or long-run can directly influence the movement of a large section of stocks in the market.

#### Highly influential in sector-specific network

In the sector wise influential strength analysis, each sector behaves very differently, and each has its unique reasons for influential rankings. However, the predominant nature of the influence of a stock on the set of stocks belonging to the same sector is because of basically three reasons. The first factor responsible for these patterns is the common industrial linkage. Generally, the stock price of the companies belonging to the same industrial segment will have a certain degree of co-movement. This is because companies in the same industry are affected by the same market conditions. Nevertheless, occasionally we can observe that the stock price of a company belonging to a common industrial segment will boost up because of disappointing news about the competitors especially in cases where the companies are competing for the same market segment. For instance, ITC Ltd benefited from bad news about the Nestle product ‘Maggi’ noodles.

The second factor responsible for these influence relationship patterns is the common impact of domestic and international macroeconomic changes in the prices of stock belonging to the same sector. The domestic and international macroeconomic changes may include a shift in Interest rates, economic outlook, and export conditions, price dynamics of commodities in international markets, inflation, deflation, currency rates and political shocks. This is evident in Technology sector, FMCG sector, and Consumer durables sector. In such cases, there is an absence of a clear dominant player throughout their ten years period.

The third factor responsible for these influence relationship patterns among stocks belonging to same sectors are vertical integration in the supply chain. One industry’s finished goods will be the raw materials or semi-finished goods for the next industry. In this context, a performance dip in the financials of the company responsible for the final product in the supply chain will result in the subsequent impact on all the other companies or sectors feeding that supply chain. This is evident in the influential stocks of Energy Sector, Textiles sector, Chemical sector and Automobiles sector.

Another aspect that needs attention is that we cannot understand the influence of a stock in a sector in isolation of the affiliated sector alone because of the fact that the Indian economy is highly integrated and shocks & stimulus of one sector can easily get transmitted to other sector specific stocks. Moreover, as finance and banking are the key backbones of an economy so any distress in this organizations can have a large scale transmission throughout the network.

## Conclusions

We start our exploratory analysis with some interesting questions concerning the influence relationship framework existent in the Indian equity market. Here, we discuss some of the key findings that we have obtained from our analysis for the queries asked before. The first set of questions that we asked were regarding the appropriate quantitative measure of influence between two stocks. From our analysis, we observe that market capitalization based influence strength gives satisfactory rankings to the stocks; hence we consider it as the right measure of quantification of an inter-stock influence relationship. The second set of question was regarding the set of influential stocks that have exhibited dominance in the Indian equity market for past 10 year’s period (2005–15). From our analysis, we could identify that stocks belonging to Reliance Industries, ICICI Bank Ltd., State Bank of India, Axis Bank Ltd., Kotak Mahindra Bank Ltd., and Larsen & Toubro Ltd. had a continuous dominance over the Indian equity market in past ten years. Also, we could characterise from the temporal evolution patterns that the magnitude of influence is very dynamic and is aligned to the favourable or unfavourable macroeconomic changes affecting the individual stocks or the industry segment they operate. As seen in the temporal plot, no stock could dominate the entire market over ten years; noticeable highs and lows were owing to the macroeconomic changes which were subsequently reflected in their company’s current & expected business performances. The third set of questions regarding the influential stocks and influential industry segments has been elaborated in the results and discussion sections of the sector-wise influence strength analysis. In this analysis, we could observe that some of the sectors like automobiles, chemicals, communication and energy had their industry-wise influence relationships pattern derived from vertical integration linkage in their sector of operations. For some of the sectors such as Textiles, FMCG, Technology, and Consumer durables, we could observe that they had their industry-wise influence relationships derived because of the common effect of either industry specific or non-specific domestic/international macroeconomic forces affecting all these sectors as a whole. We could not attribute any macroeconomic reasoning for the industry-wise influence relationships existent in some of the sectors such as the Services and Healthcare sector.

In brief, there are two key findings from the study: (i) the set of influential stocks listed on the Bombay Stock Exchange and its temporal variation over ten years; and (ii) the set of influential stocks and industry segments in each sector. These influential stocks so identified are sensitive key points of systemic risk spread in the context of Indian equity market. Any security having a high correlation structure with these influential nodes will have a greater possibility of getting impacted by the systemic shocks hitting the Indian market having its origination from any country specific, continent specific or global events. Today, systemic shocks can arise from different unrelated events which include terrorist attacks; wars and conflicts; infectious disease spread or epidemics; crude oil prices fall; economic downturns in countries such as China, US, Japan, UK and European Union members; refugee problems in Europe and UK; and Credit crisis of big multi-national banks and Financial Institutions. For a portfolio manager, handling a diversity of securities such as Mutual Funds, Pension Funds, Hedge Funds, and Derivatives; managing the impact of systemic risk originating due to these uncontrollable events in domestic and international space is a big challenge. Particularly in the case of sector-specific mutual funds management, the challenge is much magnified. In this context, the identification, characterisation, and quantification of influence relationships in individual investable sectors can reveal key actionable insights for better systemic risk management.

In addition to this key findings, some of the fundamental analysis performed in this study includes, investigation of scale-free and power law properties of construction sector; and, threshold filtering and MST analysis of cement stocks network for deriving robust correlation structures.

The study is novel in many dimensions. This is the first study to characterize influence relationships existent in equity network at three hierarchical levels. Two empirical studies, one in Brazilian equity market [63] and another in South African equity market [64] have identified the influential sectors & their temporal variation with respect to the time-frames of the financial crisis in Aug 2008. However, none of these studies, unlike our study have identified, analysed and quantified the influential stocks and industry segments operating within each of the sectors.

Analytical studies as this one could be well employed in the direction of systemic risk management. A specific application could, for instance, identify and feasibly mollify system risk spread in portfolios of broad-based, index based and sector-specific mutual funds. To such objective, there is some additional level of research that needs to be done in the future. One such feasible direction is to generate techniques and measures of how to feasible insulate the securities constituting a given portfolio from either the direct linkage or the linkage due to small-world effect to the influential nodes in an equity market. In our future work, we plan to develop econometric models for quantifying the forces responsible for correlation structures of stocks in the selected industries.

## Supporting Information

S1 Fig Mean influence strength of the constituting industry segments of Automobiles sector, Consumer Durables sector, Communication sector, and Chemicals sectors. The mean influence strength is of each stock is computed across 100 observations, and the average values of the mean influence strength of the constituting stocks of a given industry segment is the Mean influence strength of the industrial segment.
(PDF)

S2 Fig Mean influence strength of the constituting industry segments of Energy sector, Engineering sector, Financial sector, and FMCG sector. (PDF)

S3 Fig Mean influence strength of the constituting industry segments of Healthcare, Metals, Services, Technology, and Textiles. (PDF)

S4 Fig Mean influence strength of top 5 ranked stocks in Automobiles sector, Consumer Durables sector, Communication sector, Chemicals sector, and Energy sector. The mean influence strength is of each stock is computed across 100 observations, and the stocks are ranked based on this measure.
(PDF)

S5 Fig Mean influence strength of top 5 ranked stocks in Engineering sector, Financial sector, FMCG sector, Healthcare sector, and Metals sector. (PDF)

S6 Fig Mean influence strength of top 5 ranked stocks in Services sector, Technology sector, and Textiles sector. (PDF)
