# Supplementary figures and images for: Investigating the Influence Relationship Models for Stocks in Indian Equity Market: A Weighted Network Modelling Study
Source: PLoS One. 2016 Nov 15;11(11):e0166087. doi: 10.1371/journal.pone.0166087 (PMC5113066; doi:10.1371/journal.pone.0166087)

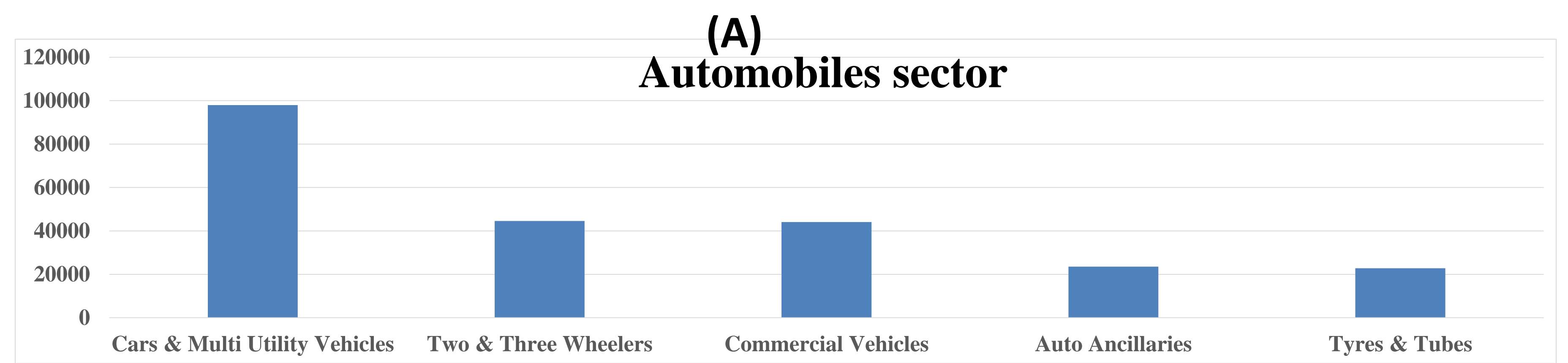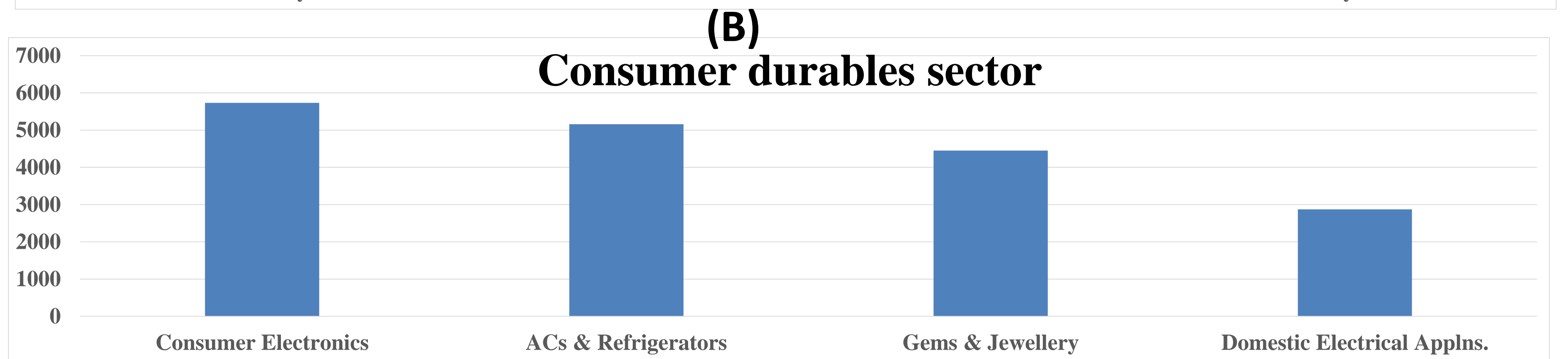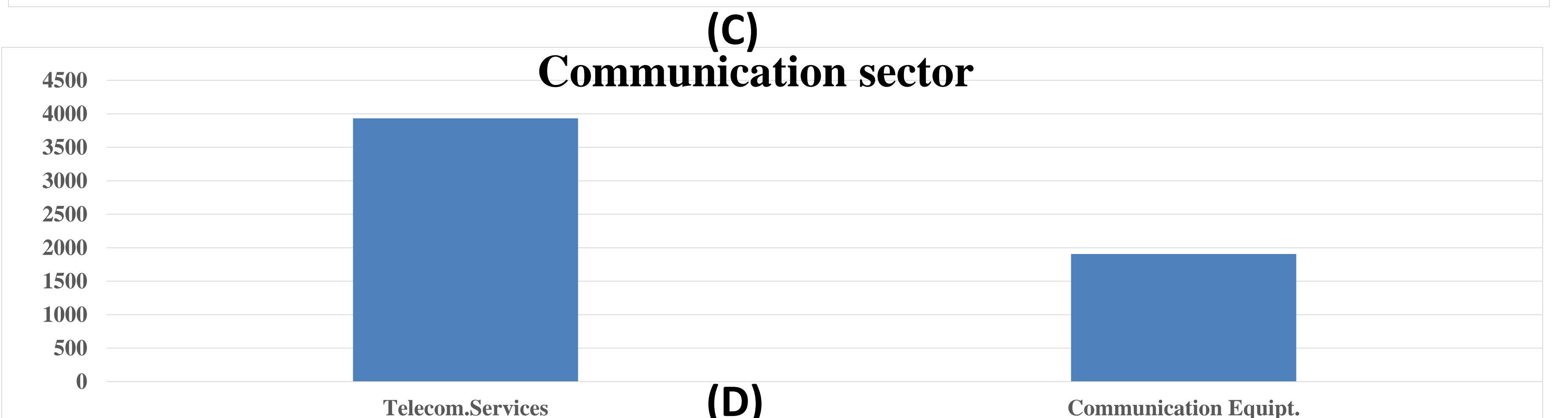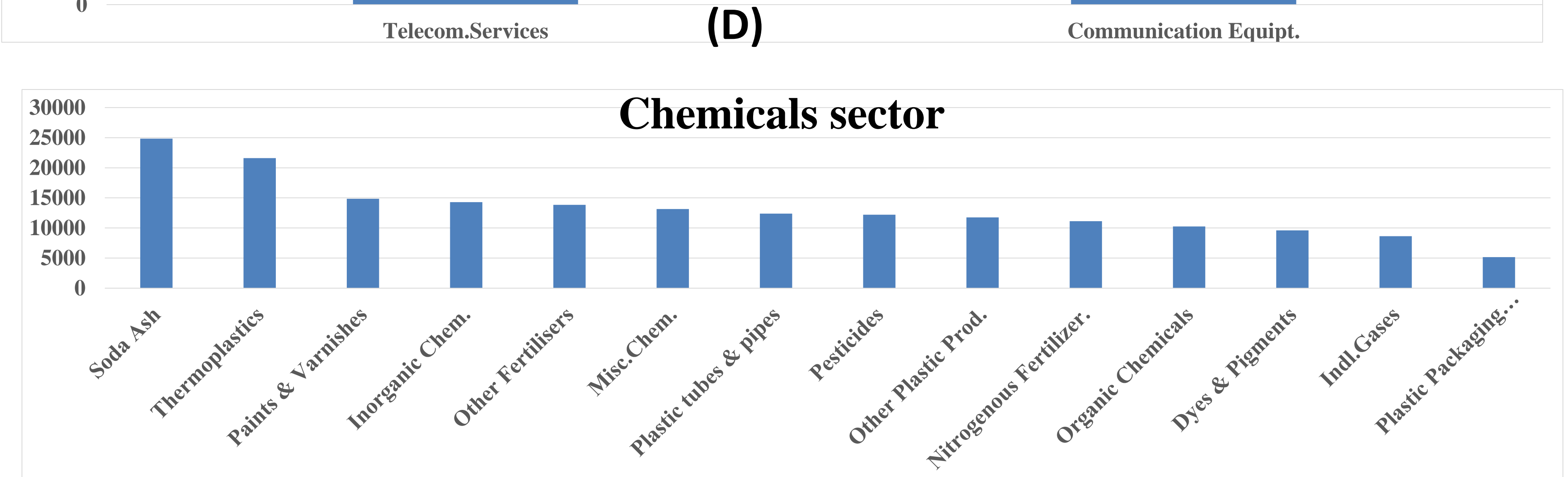

Supplement: S1 Fig — The mean influence strength is of each stock is computed across 100 observations, and the average values of the mean influence strength of the constituting stocks of a given industry segment is the Mean influence strength of the industrial segment. (PDF) [file pone.0166087.s001.pdf]

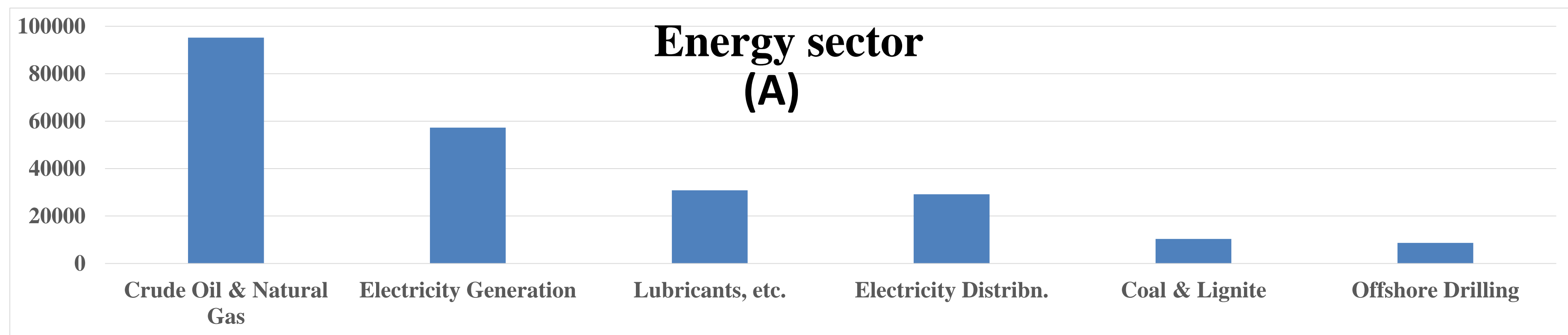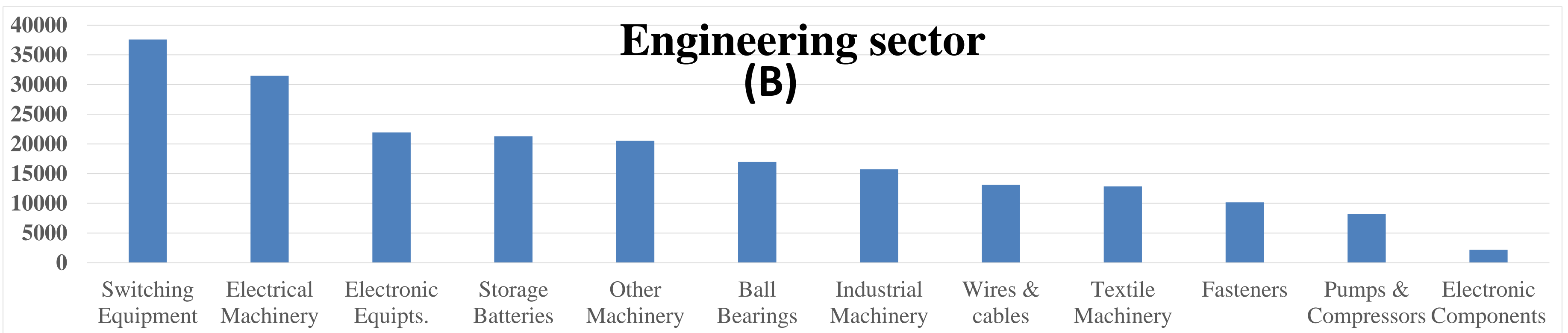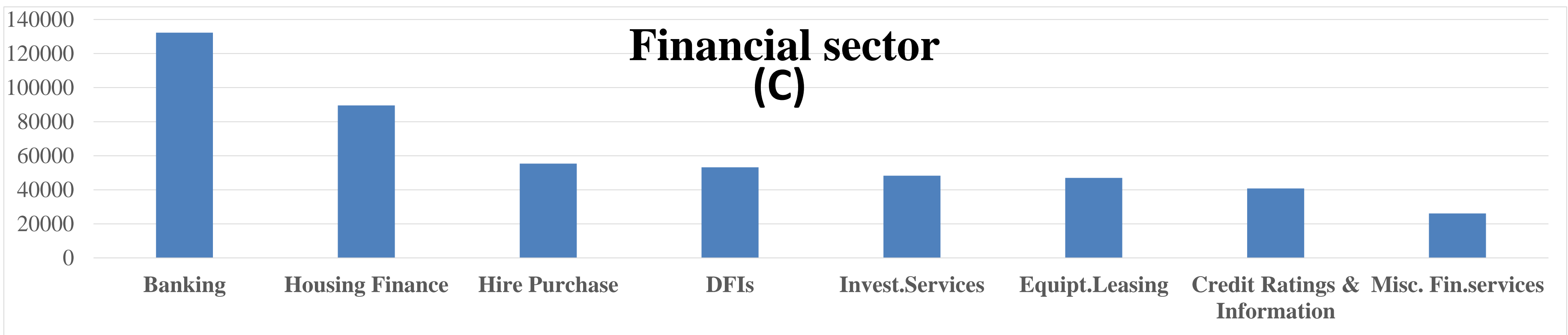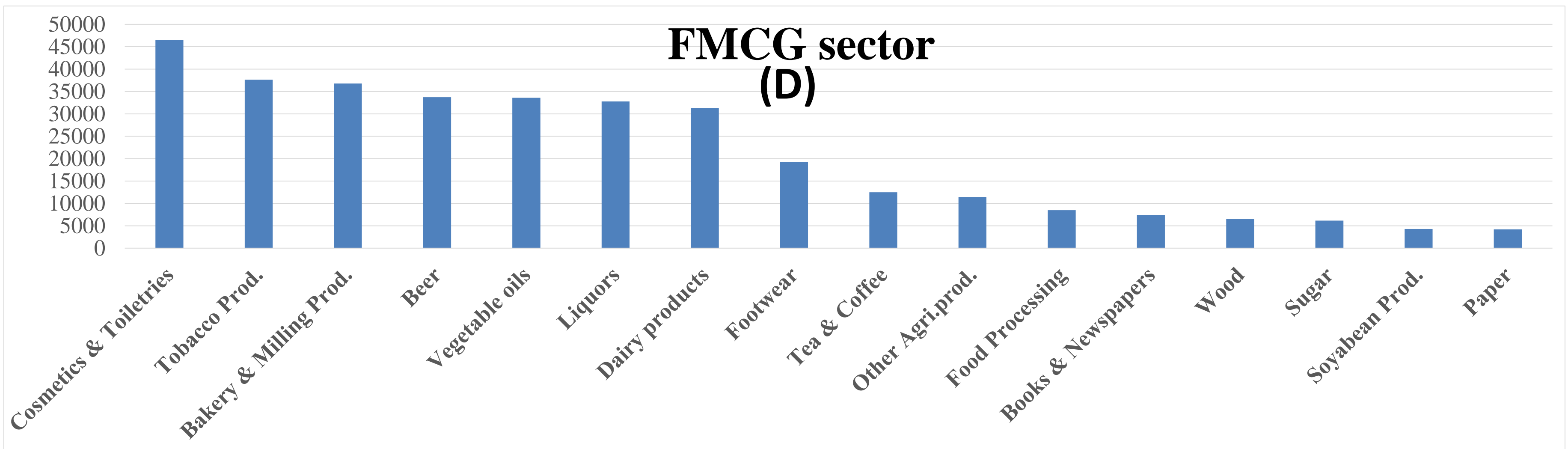

Supplement: S2 Fig — (PDF) [file pone.0166087.s002.pdf]

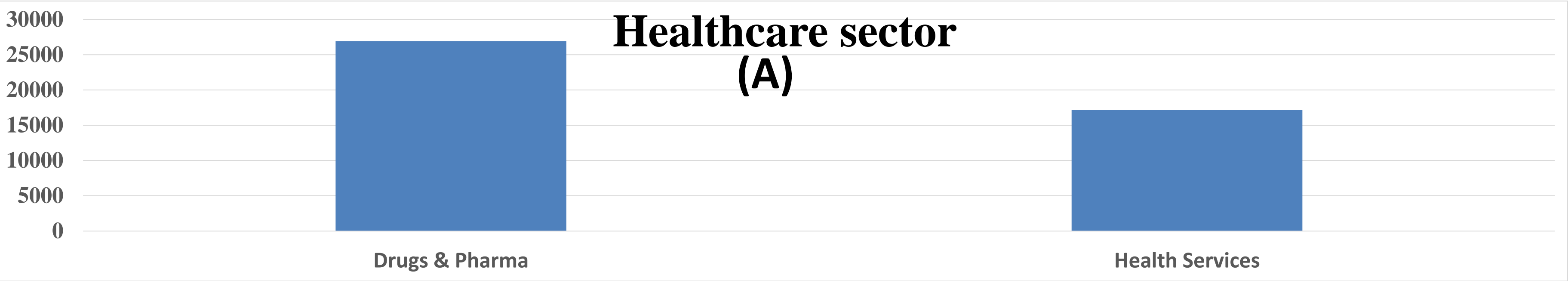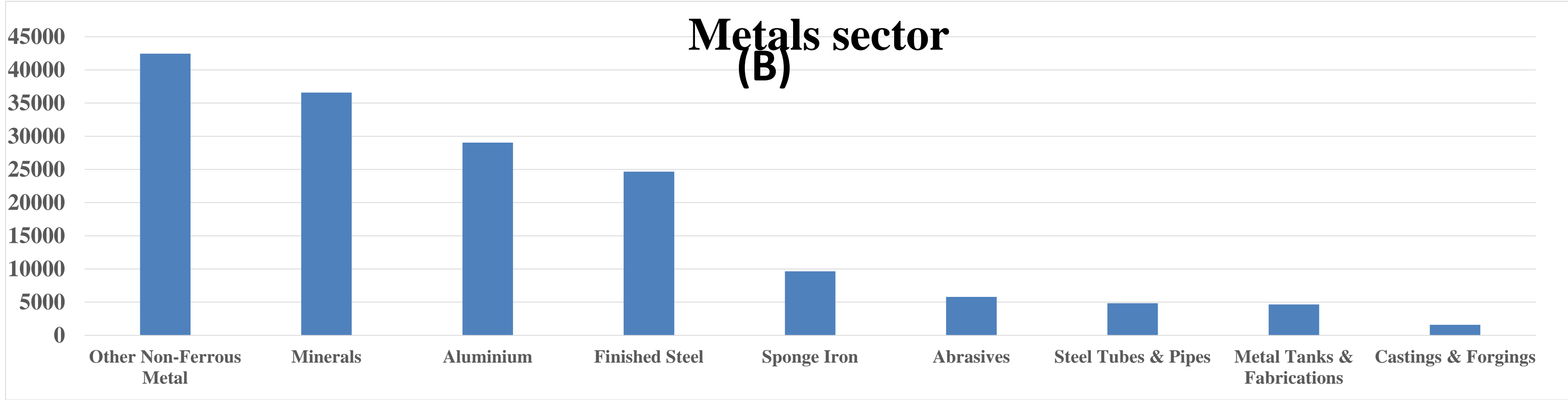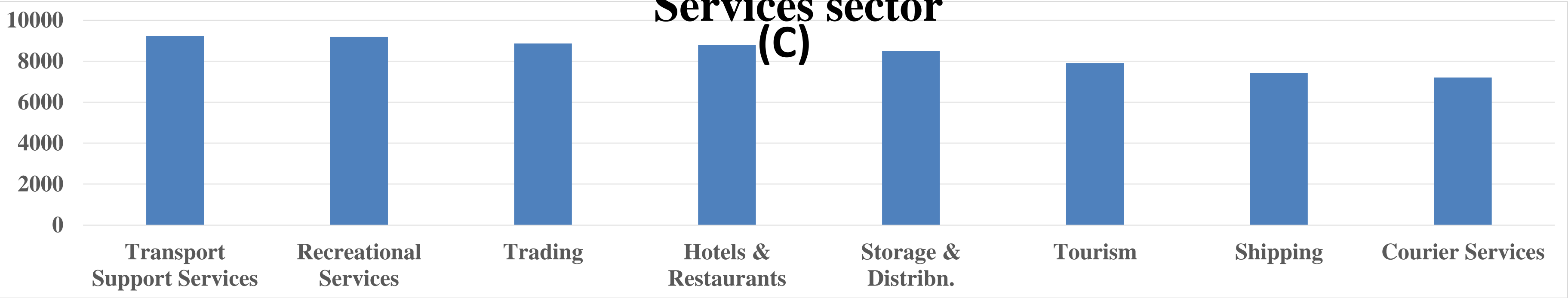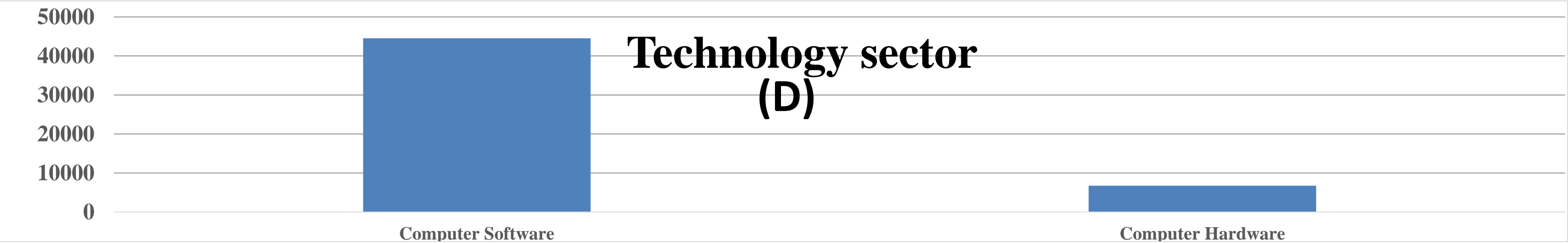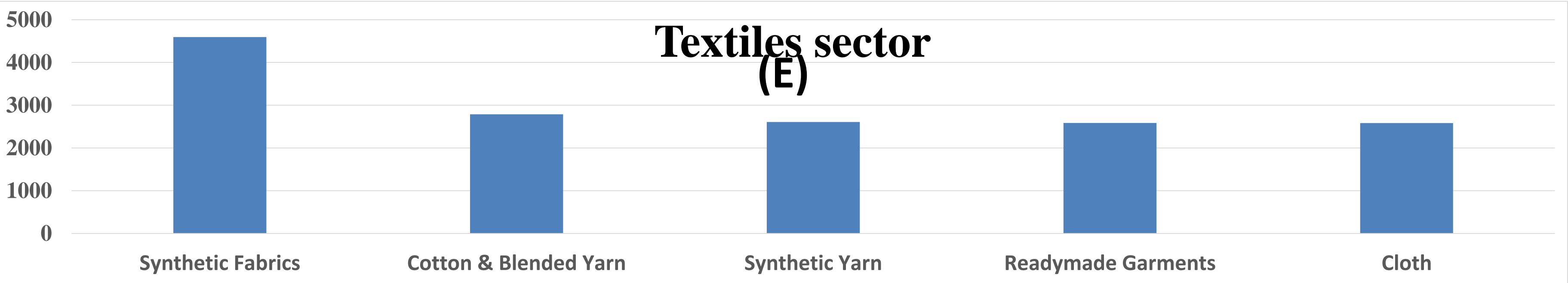

Supplement: S3 Fig — (PDF) [file pone.0166087.s003.pdf]

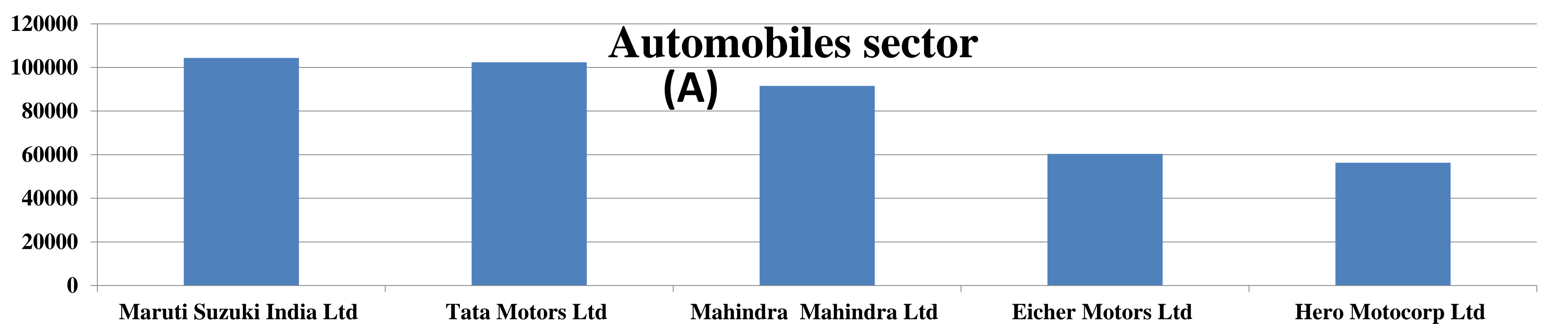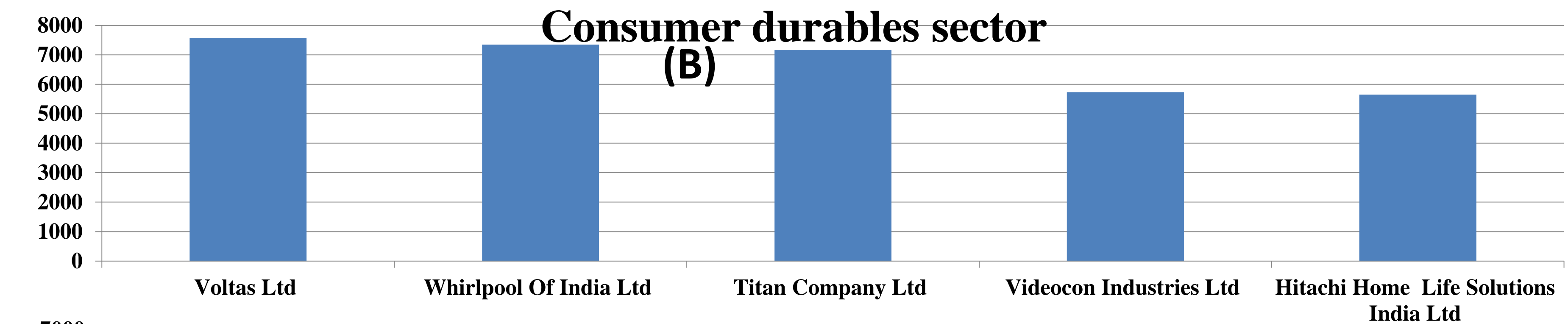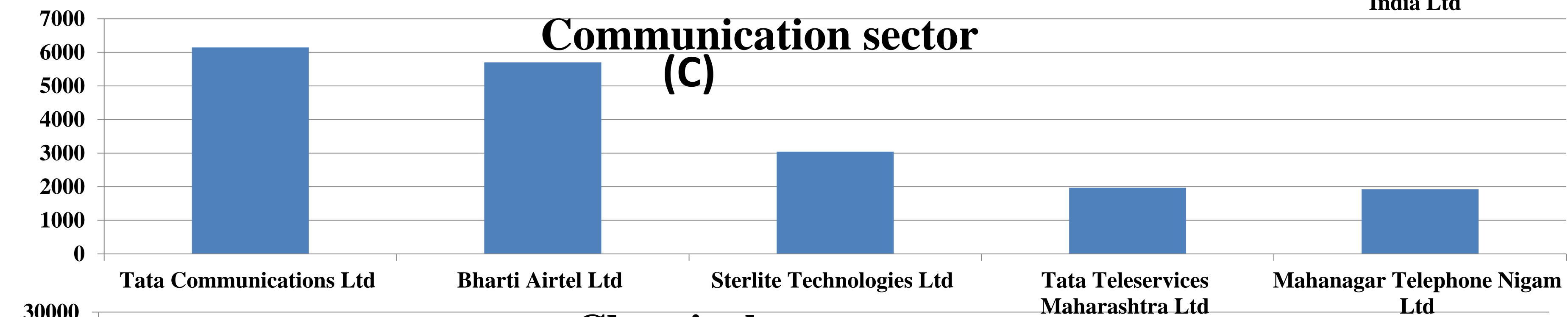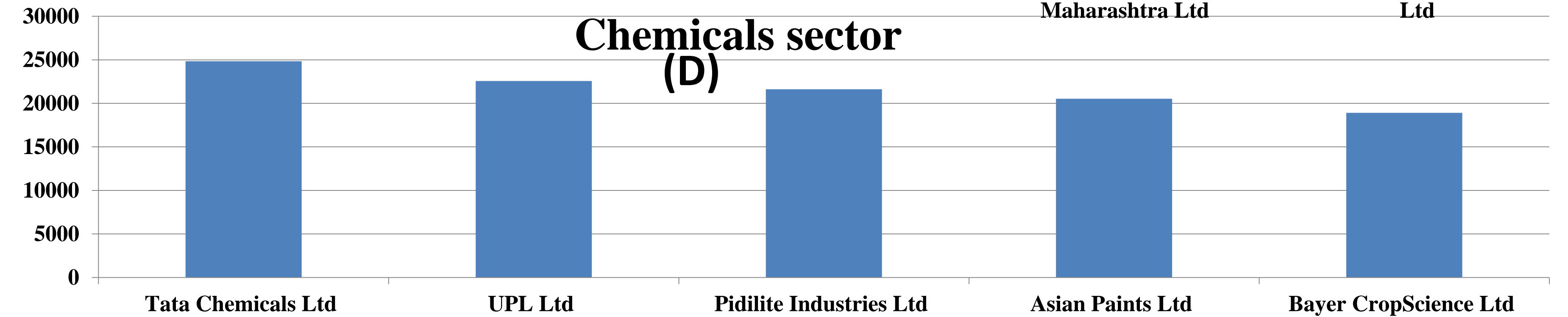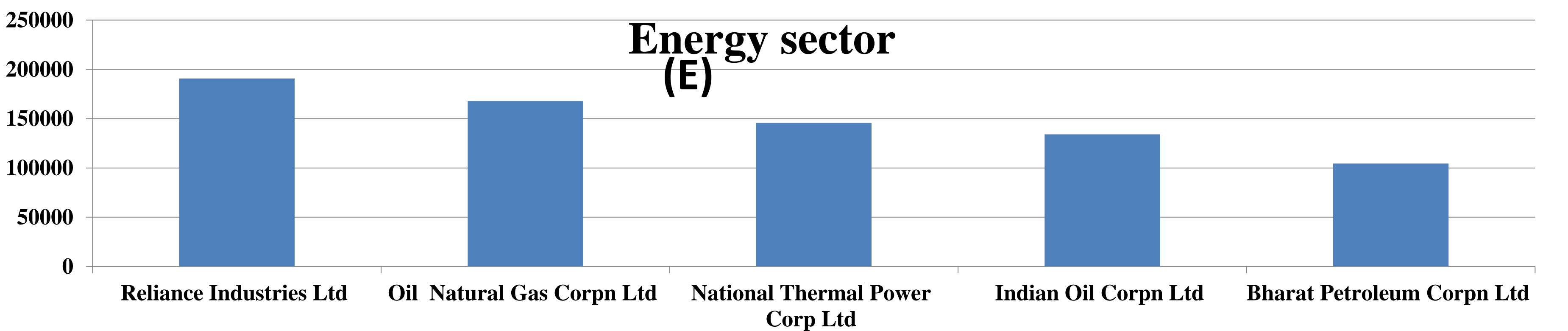

Supplement: S4 Fig — The mean influence strength is of each stock is computed across 100 observations, and the stocks are ranked based on this measure. (PDF) [file pone.0166087.s004.pdf]

**Engineering sector  
(A)**

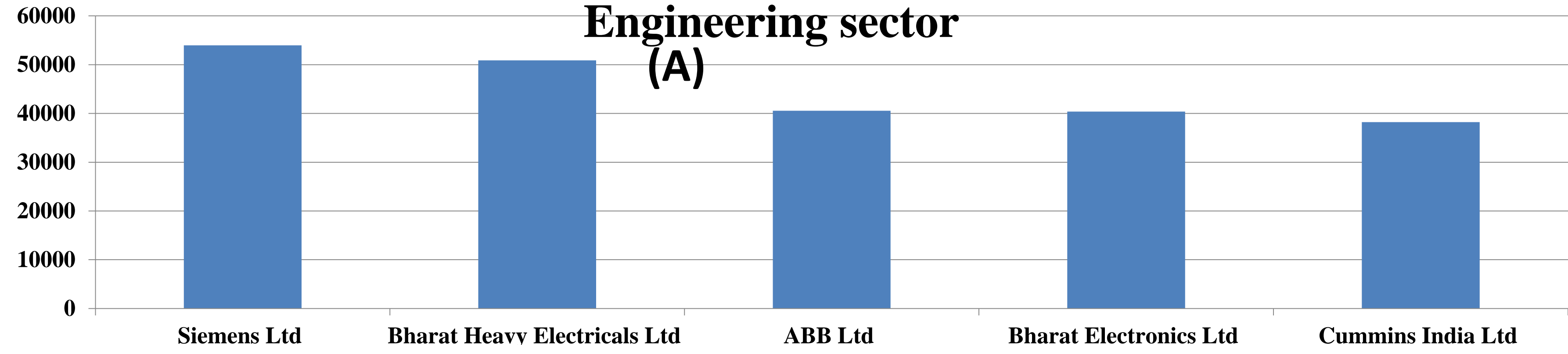

**Financial sector  
(B)**

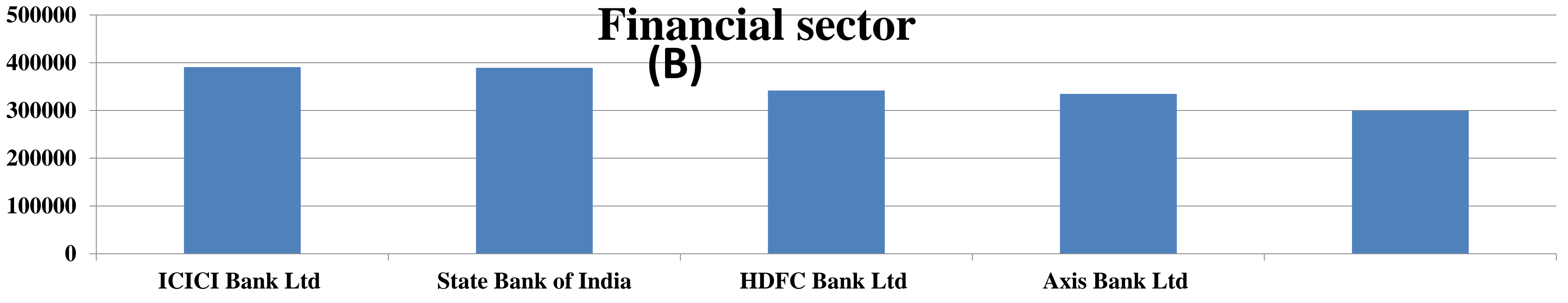

**FMCG sector  
(C)**

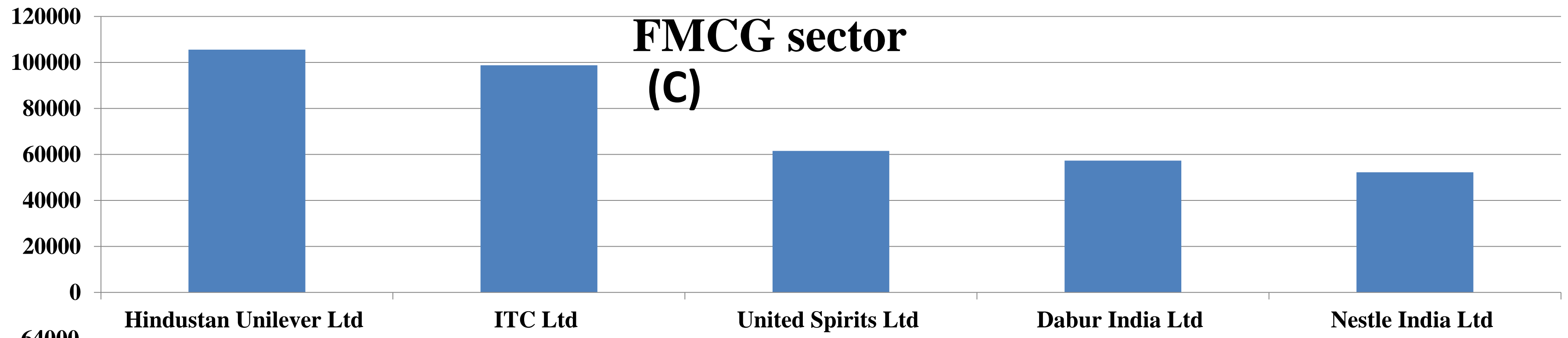

**Healthcare sector  
(D)**

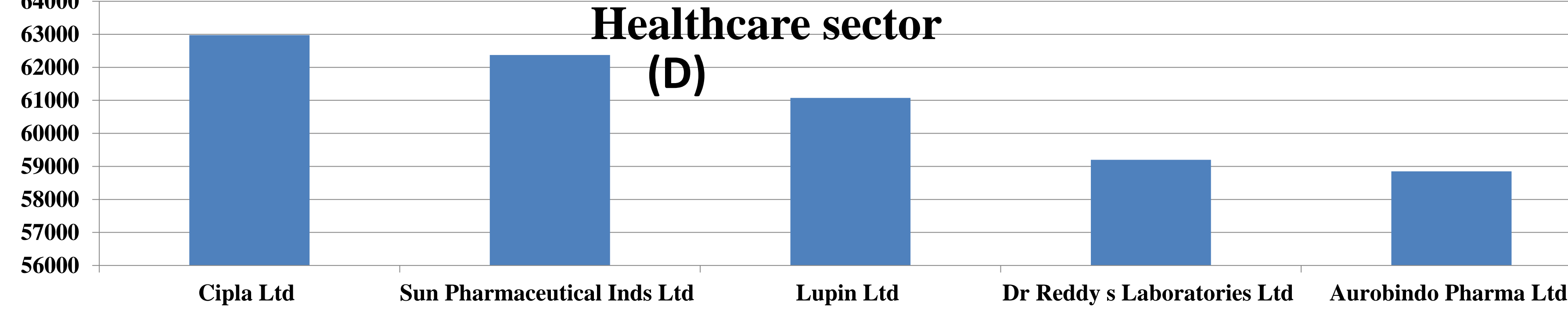

**Metals sector  
(E)**

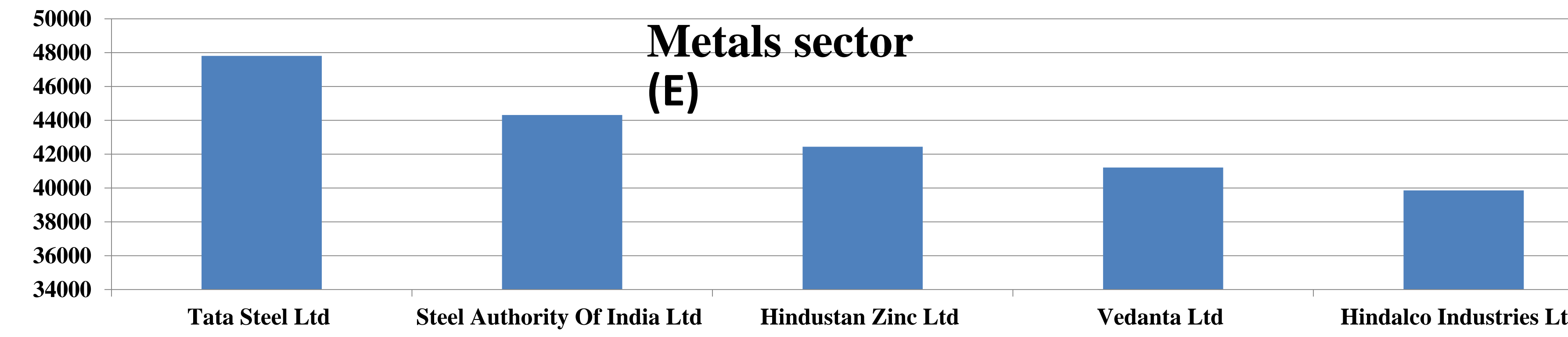

Supplement: S5 Fig — (PDF) [file pone.0166087.s005.pdf]

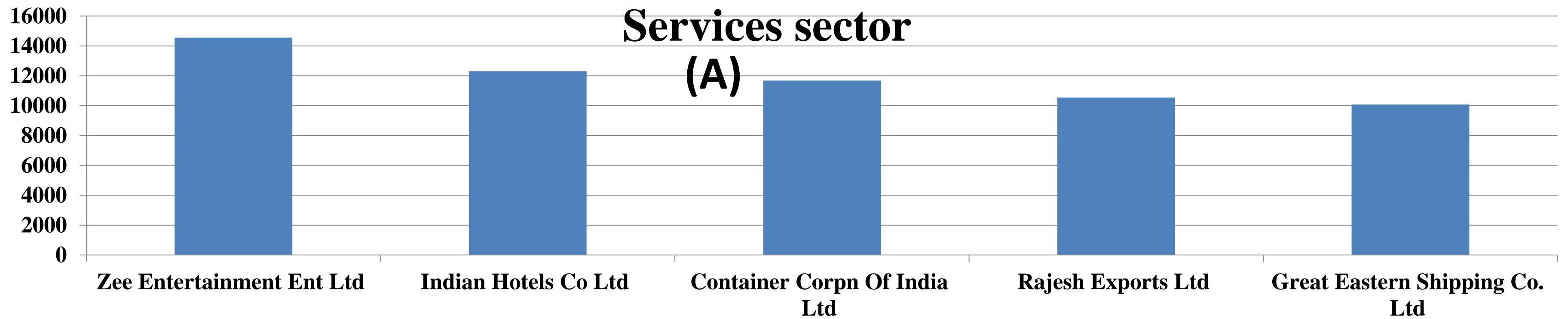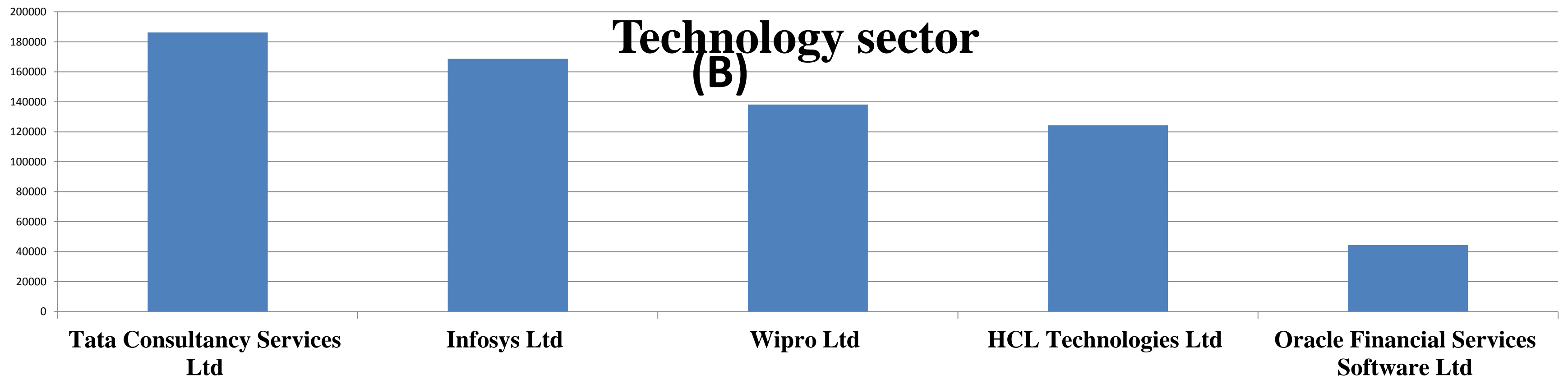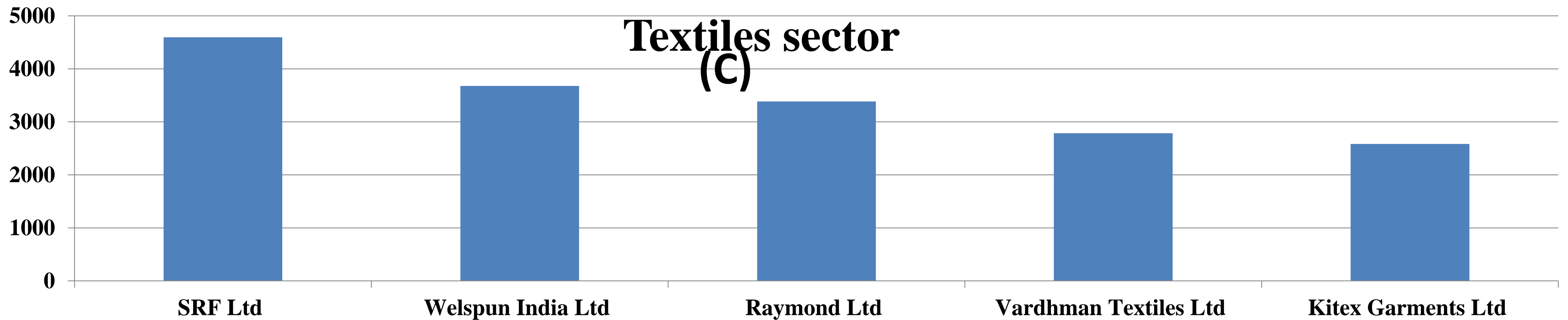

Supplement: S6 Fig — (PDF) [file pone.0166087.s006.pdf]
